# Differences in the frequency of genetic variants associated with iron imbalance among global populations

**DOI:** 10.1371/journal.pone.0235141

**Published:** 2020-07-01

**Authors:** Momodou W. Jallow, Carla Cerami, Taane G. Clark, Andrew M. Prentice, Susana Campino

**Affiliations:** 1 Nutrition Theme, MRC Unit The Gambia at London School of Hygiene & Tropical Medicine, Banjul, The Gambia; 2 Faculty of Infectious and Tropical Diseases, London School of Hygiene & Tropical Medicine, London, United Kingdom; 3 Faculty of Epidemiology and Population Health, London School of Hygiene & Tropical Medicine, London, United Kingdom; Universitat Pompeu Fabra, SPAIN

## Abstract

Iron deficiency anaemia is a major health problem affecting approximately 1.2 billion people worldwide. Young children, women of reproductive age and pregnant women living in sub-Saharan Africa are the most vulnerable. It is estimated that iron deficiency accounts for half of anaemia cases. Apart from nutritional deficiency, infection, inflammation and genetic factors are the major drivers of anaemia. However, the role of genetic risk factors has not been thoroughly investigated. This is particularly relevant in African populations, as they carry high genetic diversity and have a high prevalence of anaemia. Multiple genetic variations in iron regulatory genes have been linked to impaired iron status. Here we conducted a literature review to identify genetic variants associated with iron imbalance among global populations. We compare their allele frequencies and risk scores and we investigated population-specific selection among populations of varying geographic origin using data from the Keneba Biobank representing individuals in rural Gambia and the 1000 Genomes Project. We identified a significant lack of data on the genetic determinants of iron status in sub-Saharan Africa. Most of the studies on genetic determinants of iron status have been conducted in Europeans. Also, we identified population differences in allele frequencies in candidate putative genetic risk factors. Given the disproportionately high genetic diversity in African populations coupled with their high prevalence of iron deficiency, there is need to investigate the genetic influences of low iron status in Sub-Saharan Africa. The resulting insights may inform the future implementation of iron intervention strategies.

## Introduction

Iron deficiency anaemia (IDA) is a major health problem affecting approximately 1.2 billion people worldwide [1]. It was estimated to account for the 7^th^ leading cause of disability worldwide in 2017 [2]. IDA is regarded as the dominant cause of anaemia, accounting for approximately 60% of the global anaemia burden [3]. Pre-school children and women of childbearing age in low- and middle-income countries are the most vulnerable [3,4], particularly those living in sub-Saharan Africa, where anaemia prevalence in the general population exceeds 40% [3]. This high prevalence of IDA persists despite the existence of aggressive iron supplementation programmes for vulnerable populations (women of childbearing age and children) [5–7].

Although iron supplementation can be effective in nutritional IDA, it is ineffective in non-nutritional IDA, particularly those caused by genetic factors [8]. Therefore, the identification of the major drivers of IDA in sub-Saharan Africa is required to inform new strategies. The discovery of hepcidin and other proteins involved in iron regulation have led to the identification of genetic factors associated with altered iron homeostasis [9–11]. Several genetic variants within the iron regulatory genes have been associated with imbalances in iron homeostasis, which could lead either to iron deficiency or overload [12–16]. Genetic variants leading to excess body iron occur mainly in the haemochromatosis (*HFE*) gene but are also seen in hepcidin (hepcidin antimicrobial peptide (*Hamp*)), transferrin receptor 2 (*TFR2*), solute carrier family 40 member 1 (*SLC40A1*), haemojuvelin (*HJV*) and transferrin (*TF*) genes [9–11]. These loci have important functions in the iron homeostasis pathways. For example, hepcidin regulates iron absorption and release [17]. Genetic polymorphisms in genes involved in the hepcidin suppressive pathway such as *TMPRSS6* (transmembrane protease serine 6), have been associated with low iron status [18–20] and a condition described as iron-refractory iron deficiency anaemia (IRIDA) [18–21]. Individuals with IRIDA have a hereditary form of anaemia that does not respond to oral iron supplementation [22,23]. Although IRIDA is quite rare, it may be at the extreme end of a broad continuum of disease, since *TMPRSS6* genetic variants can lead to different degrees of iron deficiency and anaemia [18–20]. In addition, SNPs in the *TF* gene, also important in iron transport to cells, have also been reported to affect iron status and lead to low iron status [24–26]. Furthermore, SNPs in the divalent metal transporter 1 (*DMT1*), the duodenal apical iron transporter encoded by the *SLC11A2* gene have been associated with an unusual syndrome characterized by microcytic anaemia and a paradoxical iron overload [27,28].

A genome-wide association study (GWAS) investigating genetic determinants of relevant haematological traits and iron status have identified variants in *TF* and *HFE*, which explain approximately 40% of variation in serum transferrin levels [26]. Also, GWASs have identified genetic variants in *TMPRSS6* associated with alterations of serum iron status, erythrocyte volume [29], and haemoglobin levels [20]. African populations have been greatly under-represented in such studies. A GWAS using an African population cohort replicated only the association of two SNPs in *TMPRSS6* with lowered haemoglobin concentration, and one SNP in *TF* with increased ferritin concentrations [30]. Differences in the frequencies of risk alleles and linkage disequilibrium patterns might explain the limited replication of association results between European, Asian and African populations. Hence, there is a need to investigate population-specific genetic variants that may affect iron status.

Here, we conducted a review of the literature to identify genetic variants that have been associated with iron imbalances, with a special focus on SNPs in *TMPRSS6*, *HAMP*, *TF*, *TFR2*, *SLC40A1* and *HFE* genes. We investigated the geographical distribution of studies and assessed the differences in allele frequency of these polymorphisms and their linkage disequilibrium patterns across global populations. We use genetic data from our Keneba Biobank in rural Gambia and from the 1000 Genomes Project. We also explored the possibility of natural selection acting on these genes and any resulting population-specific selection, as measured through large differences in allele frequencies between geographic regions. As part of this, we sought to summarize the geographical distribution of genetic determinants of iron status. The resulting insights may assist in designing future genetic association studies that are geared towards identifying population-specific genetic risk factors affecting iron status and, ultimately, guiding population-specific iron intervention strategies.

## Methods

### Selection of SNPs

A literature search was conducted using the Human Genetic Epidemiology (HuGE) navigator, a database of published population-based human genetic epidemiology studies. This review was complemented using the PubMed site with search terms: “anaemia”, “iron”, “iron overload”, “iron deficiency anaemia”, “iron imbalance”, “hepcidin”, “genome-wide association study”, “GWAS”, “haematology traits”, and “haemochromatosis”. The search was conducted on articles published between 01 January 1999 to 31 October 2018. The assessment process included examining titles and abstracts of studies and excluding duplicates. Articles were included if they were: (1) original research papers conducted in humans; (2) tested for an association between at least one SNP in the genes commonly linked to dysregulated iron status (*TMPRSS6*, *HAMP*, *TF*, *TFR2*, *SLC40A1* and *HFE*) or iron status measures. These include iron status biomarkers (serum iron, transferrin, ferritin, soluble transferrin receptor, transferrin saturation, total iron binding capacity, unsaturated iron binding capacity and hepcidin) alone or in combination with haematology traits (haemoglobin, red blood cells, hematocrit, mean corpuscular haemoglobin and mean corpuscular hemoglobin concentration). Animal studies, case reports, commentaries and articles not written in English were excluded. Rare variants reported in a single individual or family were discarded. Information on genomic and gene location, allele ancestry, minor allele variant and the predicted consequence of each SNP were obtained from the Ensembl dataset (release 98) [31] and the dbSNP nucleotide variation database [32].

### Genotype data and statistical analysis

We obtained genotype data from the Keneba at MRCG at LSHTM [33] (n = 3,116 healthy Gambian individuals) and from the 1000 Genomes project [n = 2,504; 26 populations categorised into African (AFR, n = 661), European (EUR, n = 503), American (AMR, n = 347), East Asian (EAS, n = 504) and South Asian (SAS, n = 489)] [34]. Genotyping of the Keneba Biobank populations was performed using the Infinium 240K Human Exome Beadchip (v1.0 and v1.1). Genotype calling was performed using data-driven clustering (Genome Studio, Illumina, CA, USA).

We assessed the differences in allele frequencies for SNPs with genotype calls in both the Keneba Gambian and the pan-African populations in the 1000 Genomes Project. Linkage disequilibrium (LD) measures (*D’* and *r*^*2*^) were calculated using the R package Genetics [35]. The correlation between minor allele frequencies across populations was calculated using the Pearson’s correlation coefficient in the R package corrplot.

We calculated the allele risk score for each individual by aggregating the number of risk alleles an individual carried. To do this, from each SNP, the risk allele was assigned 1 and alternate allele assigned 0. For the genotype of each SNP, an individual was given either 0 (wildtype), 1 (heterozygote) or 2 (homozygote for the risk allele). Using this information, we determined the allele risk scores across populations for both low and high iron SNPs. For 23 SNPs it was not possible to identify the associated alleles (e.g. just a “A/T” label) or classify the direction of association (e.g. absence of regression coefficients). Also, for some SNPs (*TF* rs3811658 and rs1880669, and *TMPRSS6* rs2072860 and rs2111833) (S1 Table) we found contradictory information about their association with iron biomarkers between studies. They were all excluded from risk allele analysis. Statistic differences in the distribution of risk alleles between populations were calculated using a Wilcoxon rank sum test in the R statistical package [36]. To allow for multiple comparisons, a Bonferroni correction was applied.

The minor allele frequency (MAF), observed and expected heterozygosities and measures of population differentiation (global and pairwise *F*_ST_ to assess differences in allele frequencies) were calculated from the genotype data for all iron-associated SNPs using a combination of the R packages Adegenet [37], Hierfstat [38] and Pegas [39]. Weir & Cockerham *F*_ST_ values were calculated and range from 0 to 1, where a zero value implies that the two populations are interbreeding, and a value of one means that the two populations do not share any genetic diversity. Population Branch Statistic (PBS) values were calculated using the *F*_*ST*_ data from the comparison of three populations (AFR-EUR, AFR-SAS, EUR-SAS) according to methods described elsewhere [40]. To evaluate the significance of the observed *F*_ST_ and PBS values, the results were compared with the empirical distribution of genome-wide SNPs reported by others using individuals from several geographical locations and including data from the HapMap and HGDP [41–45].

Statistical differences between MAFs were analysed using the two-proportion Z-Test in R. The integrated Haplotype Score (iHS) [46,47] statistic was investigated using Haplotter (http://haplotter.uchicago.edu/) [48] and HGDP selection browsers (http://hgdp.uchicago.edu/cgi-bin/gbrowse/HGDP/) at the individual genes and surrounding regions.

### Ethics statement

The Keneba Biobank Project received ethical approval from the MRCG at LSHTM Scientific Coordinating Committee and the MRCG at LSHTM/ Gambia Government Joints Ethics Committee (SCC1185). Written informed consent was obtained from each participant.

## Results

### Genetic variants associated with iron imbalances

**Fig 1:**
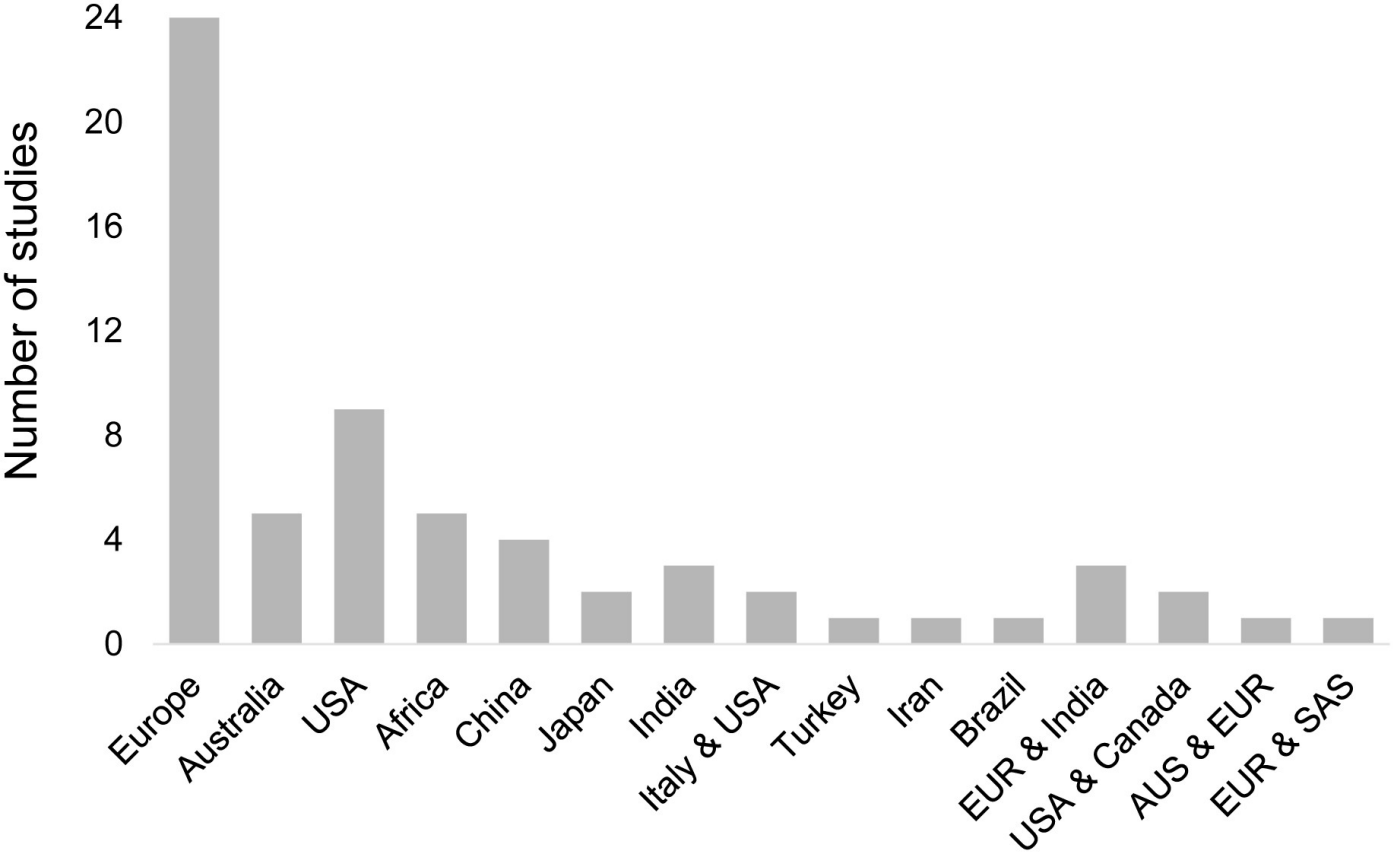
Geographical locations of the sixty-four studies that reported genetic variants associated with iron imbalance. Nine studies involved multi-ethnic populations. AUS, Australia; EUR, Europe; SAS, South Asia.

A total of 64 studies were selected that contained data on the effects of genetic polymorphisms on the variations in iron or haematological parameters (S1 Table). The majority of the studies (59/64) were conducted in Europe, Asia and the USA (Fig 1, S2 Table). Only five studies were conducted in Africa, two in Rwanda [49,50], one in Zimbabwe [51], one in South Africa [52] and one meta-analysis across Kenya, Tanzania and South Africa [30]. Across the 64 studies, 50 SNPs were identified in six genes (*TMPRSS6*, *HAMP*, *TF*, *TFR2*, *SLC40A1* and *HFE)* (S1 Table). More than half of these SNPs were found to be associated with variation in iron or in other haematological parameters in more than one country (29 SNPs, 58%). Of these 29 SNPs, 79.3% were reported in more than one ethnic group (S2 Table). Nine SNPs lead to a missense mutation causing an amino acid change, four SNPs had synonymous variants, and the remaining SNPs are in intronic (n = 32), regulatory or intergenic regions (n = 5).

The highest number of SNPs were identified in the *TMPRSS6* gene region (n = 23), where the majority were associated with IRIDA, iron deficiency or indicators of low iron status (S1 Table). The most commonly reported *TMPRSS6* SNP was rs855791, followed by rs4820268, rs2235321 and rs2235324, all associated with biomarkers of low iron status. These SNPs have been mainly reported in non-African populations. Three *TMPRSS6* SNPs (rs5756504, rs5756506 and rs1421312) were also associated with biomarkers indicating elevated iron status (S1 Table).

The *TF* gene had the second highest number of SNPs related to either low or high iron status (n = 18). The most common of these (rs3811647) was reported by ten studies (S1 Table). This variant has been mainly associated with elevated transferrin and total iron binding capacity levels [26,53,54]. For the *SLC40A1* gene, three SNPs were selected that led to alterations in iron status measures and severity of haemochromatosis [50,51,55–57]. One SNP was identified in *HAMP* (rs10421768) [30,55,58–60] and one in *TFR2* (rs7385804) (6,7,33,34,37,38), both of which were found to be associated with increases in haemoglobin and alterations serum in ferritin concentrations [30,55,56,58,61–63]. For the *HFE* gene, we found four SNPs that have been associated with alterations in haemoglobin and/or an increase in the genetic risk of hereditary haemochromatosis [13,14,19,20,24,26,29,56,62,64–74]. The most commonly reported *HFE* variant is rs1800562 (C282Y) [13,19,24,26,29,71,72], which has been widely associated with the severe form of hereditary haemochromatosis in European descents.

### Global geographic distribution of allele frequencies

**Fig 2:**
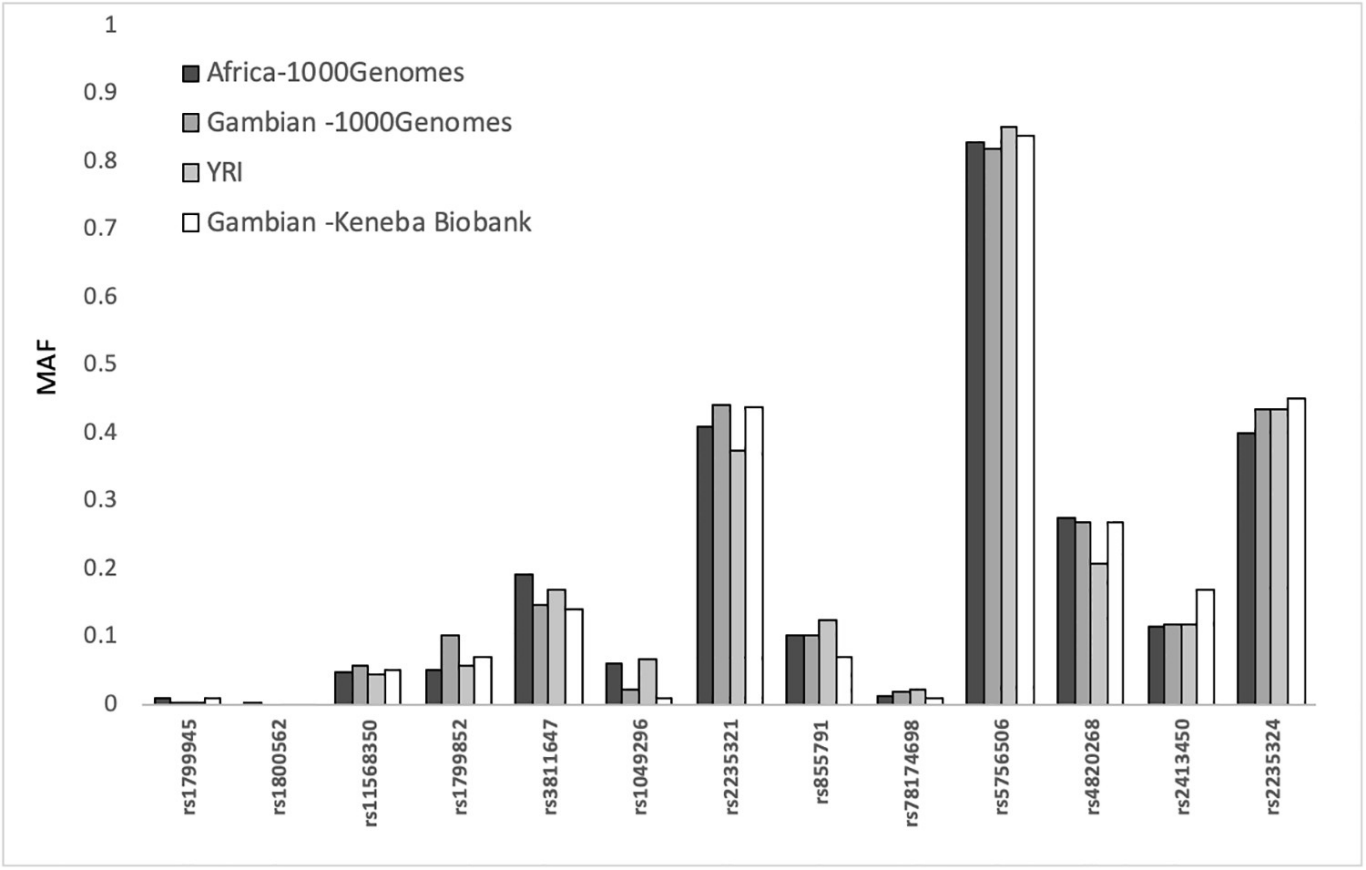
Minor allele frequencies (MAF) of 13 SNPs across African populations. Comparing MAF between the two Gambian datasets, Yoruba (YRI) from Nigeria and overall African populations included in the 1000 Genomes Project. The minor alleles were defined by the 1000 Genomes Project.

We investigated the allele frequencies of the 50 SNPs across data from the Keneba Biobank at the MRCG at LSHTM in The Gambia (n = 3,116) and the 1000 Genome project (n = 2,504) [34]. The 1000 Genomes project includes data from African (AFR, n = 661; including from The Gambia), European (EUR, n = 503), American (AMR, n = 347), East Asian (EAS, n = 504) and South Asian (SAS, n = 487) populations. Only thirteen of the 50 SNPs in the *TF*, *TMPRSS6*, *HFE* and *SLC40A1* genes, were available in the Keneba Biobank, because not all the SNPs were on the Exome chip that was used for genotyping this population. When we compared the allele frequencies of the SNPs with data from The Gambians in the Keneba Biobank with the pan-African populations in the 1000 Genomes project, we observed minimal differences (Fig 2).

**Fig 3:**
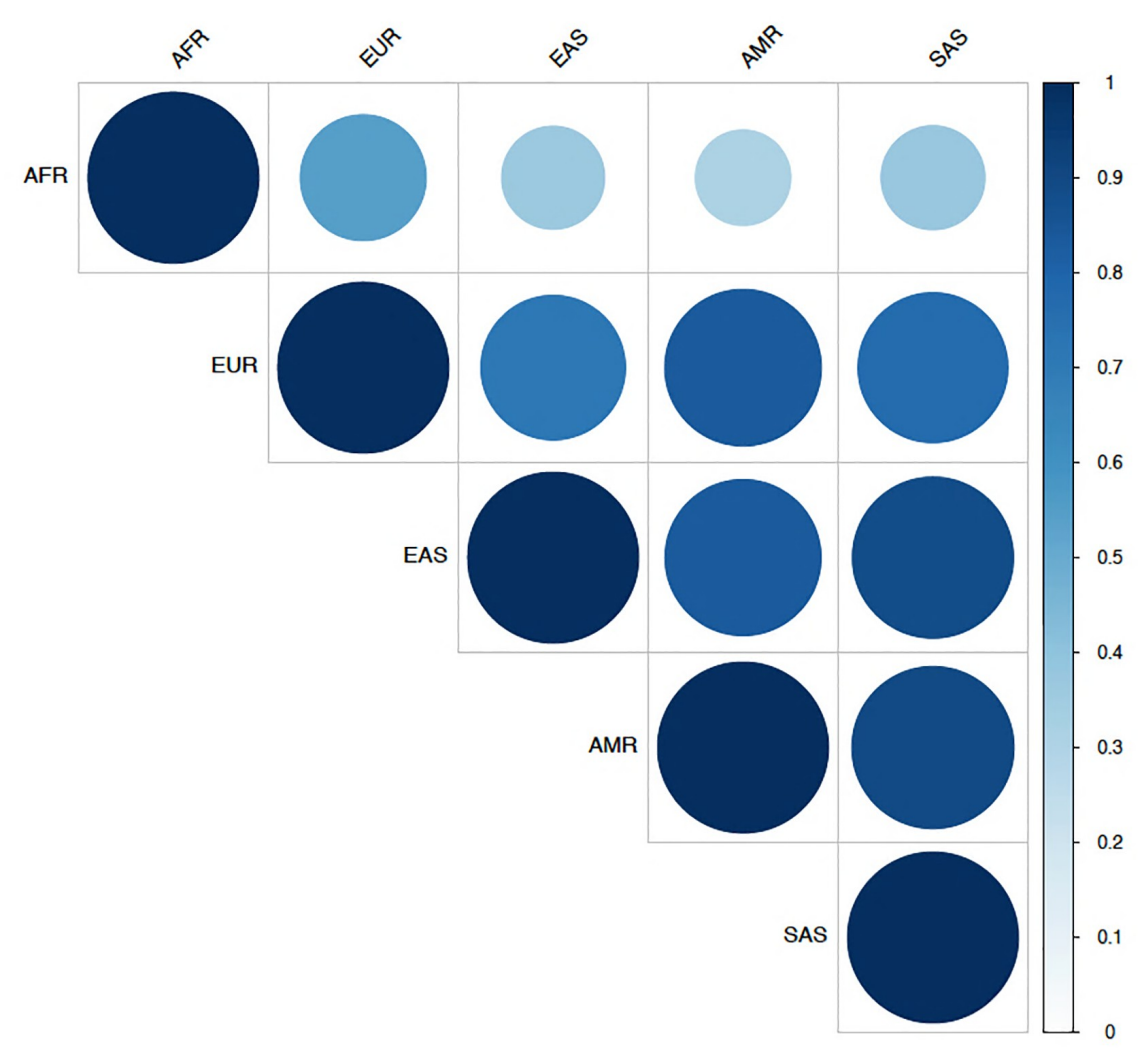
Correlation of minor allele frequencies between different geographic regions. Correlation coefficients were obtained by pairwise comparisons of each of the 50 SNPs identified across two populations. They are coloured according to the value using a gradient from white (representing 0 for no correlation) to dark blue (1 for perfect correlation). The minor allele variant was defined by the 1000 Genomes Project. AFR, African; EUR, European; AMR, American; EAS, East Asian; SAS, South Asian.

**Fig 4:**
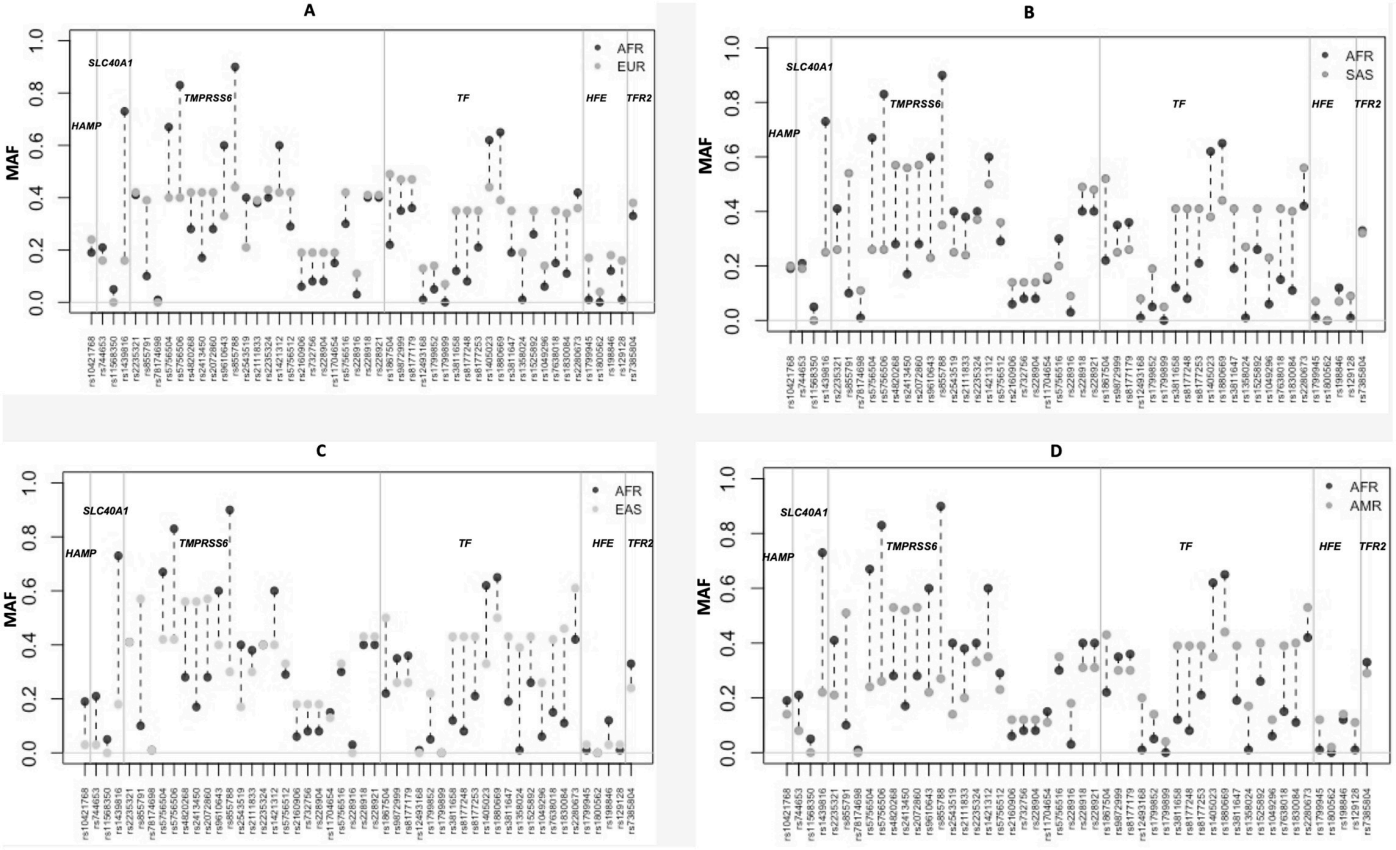
The differences in minor allele frequencies of SNPs in the six genes investigated, across different geographic regions. The comparisons were made between Africans and other global populations (A) Africa vs. Europe; (B) Africa vs. South Asia; (C) Africa vs. East Asia; (D) Africa vs. America. The thick grey lines indicated borders between SNPs in different genes: *HAMP*, *SLC40A1*, *TMPRSS6*, *TF*, *HFE* and *TFR2*. The minor alleles were defined according to the 1000 Genomes Project database [34]. AFR, African; EUR, European; AMR, American; EAS, East Asian; SAS, South Asian.

For the majority of SNPs, the MAFs in the African populations were very different to other worldwide populations (Figs 3 and 4). The greatest allele frequency differences were observed in rs1439816 in *SLC40A1*, and in several SNPs in *TMPRSS6* (including rs855791 and rs855788). The intronic variant rs1439816 in the *SLC40A1* gene has a MAF of ~20% in the non-African populations but reaches >73% frequency in Africa (S1 Table). The missense variant A736V (*TMPRSS6* rs855791) is the most reported SNP associated with iron deficiency and has a MAF of ~50% across all non-African populations, but in Africa it only reaches 10% (7% in the MRCG Keneba Biobank population) (Fig 4). The intronic variant rs855788 in *TMPRSS6* has a MAF of ~30% across non-African populations, contrasting with a frequency in excess of 86% in the African populations (Fig 4).

From the selected SNPs, several in African (n = 10 SNPs) and East Asian (n = 11 SNPs) populations have fixed ancestral alleles or low MAF (<5%) (Fig 4). These SNPs include four missense variants, with the lowest overall MAF or with fixed ancestral alleles in several populations (associated with low iron: *TMPRSS6* rs78174698 and *TF* rs1799899; associated with increased serum ferritin: SLC40A1 rs11568350; associated with haemochromatosis: *HFE* rs1800562). The *TMPRSS6* rs78174698 (P555S) MAF is low overall (<2%) across most populations, except in South Asia where the minor allele is >10%. The minor allele for rs1799899 (G277S) is rare in Africa and East Asia (<0.2%), and only reaches >4% MAF in European, American and South Asian populations. For *SLC40A1* rs11568350 (Q248H), the minor allele reaches 5% in Africans, including in both The Gambian populations in the two datasets analysed. In the other global populations, the ancestral allele is almost fixed. The variant A allele of rs1800562 (C282Y) has the highest frequency in European populations (4.3% and 5.3% in Caucasians from Europe in the 1000 Genome Project and in the HapMap CEU population, which have ancestry from Northern and Western Europe, respectively). The frequency of this variant is extremely low in Africans (0.2% in the 1000 Genomes project) and it was not detected (MAF = 0) in the Keneba Biobank population.

**Fig 5:**
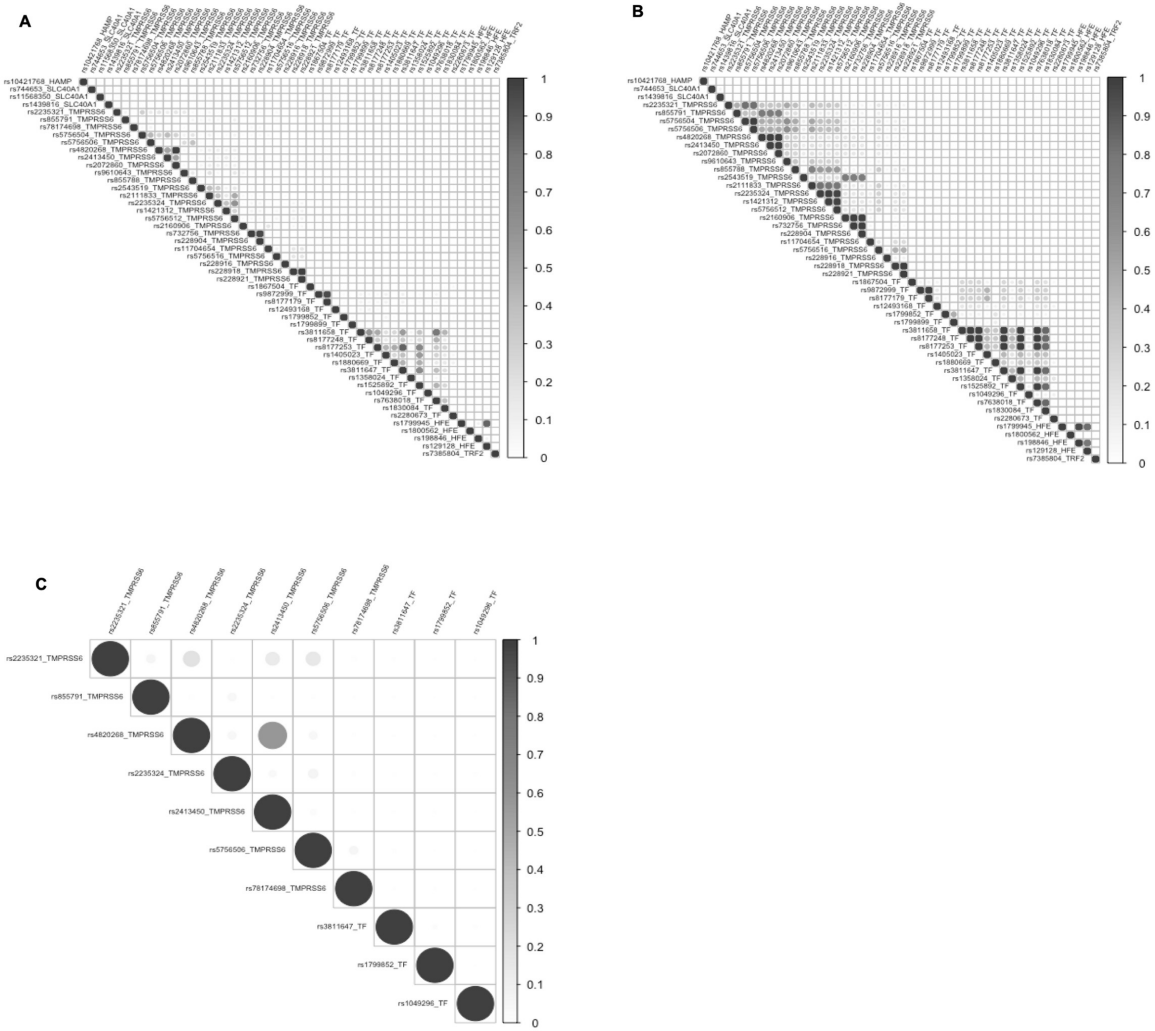
Linkage disequilibrium (LD) plots in SNPs in *HAMP*, *SLC40A1*, *TMPRSS6*, *TF*, *HFE* and *TFR2* genes. LD plot showing r^2^ values in SNPS associated with iron imbalances in: (A) African populations, (B) European populations and (C) Gambian population in the Keneba Biobank.

We also investigated the population-specific linkage disequilibrium (LD) patterns between SNPs in the candidate genes. There were blocks of high LD in the non-African population, and the overall levels of LD were lower in the African populations (Fig 5, S1 Fig), including in The Gambia. In contrast, the SNPs in the *TF* gene still showed a pattern of high LD in the African populations.

### Distribution and frequency of iron imbalance risk alleles

To investigate if any population had an over- or under-representation of risk alleles leading to iron imbalances, we first classified the alleles as protective or susceptible based on previous associations with low or high iron status or related biomarkers (S1 Table). A total of 23 SNPs were included in the risk allele analysis (see Methods for exclusion criteria). Eleven SNPs had alleles that were clearly associated with low iron, iron deficiency anaemia and/or IRIDA (SNPs in *TMPRSS6* (rs855791, rs2235321, rs2235324, rs4820268, rs2413450, rs228916, rs228918 and rs228921) and *TF* (rs3811647, rs1799899 and rs8177253) (S1 Table).

**Fig 6:**
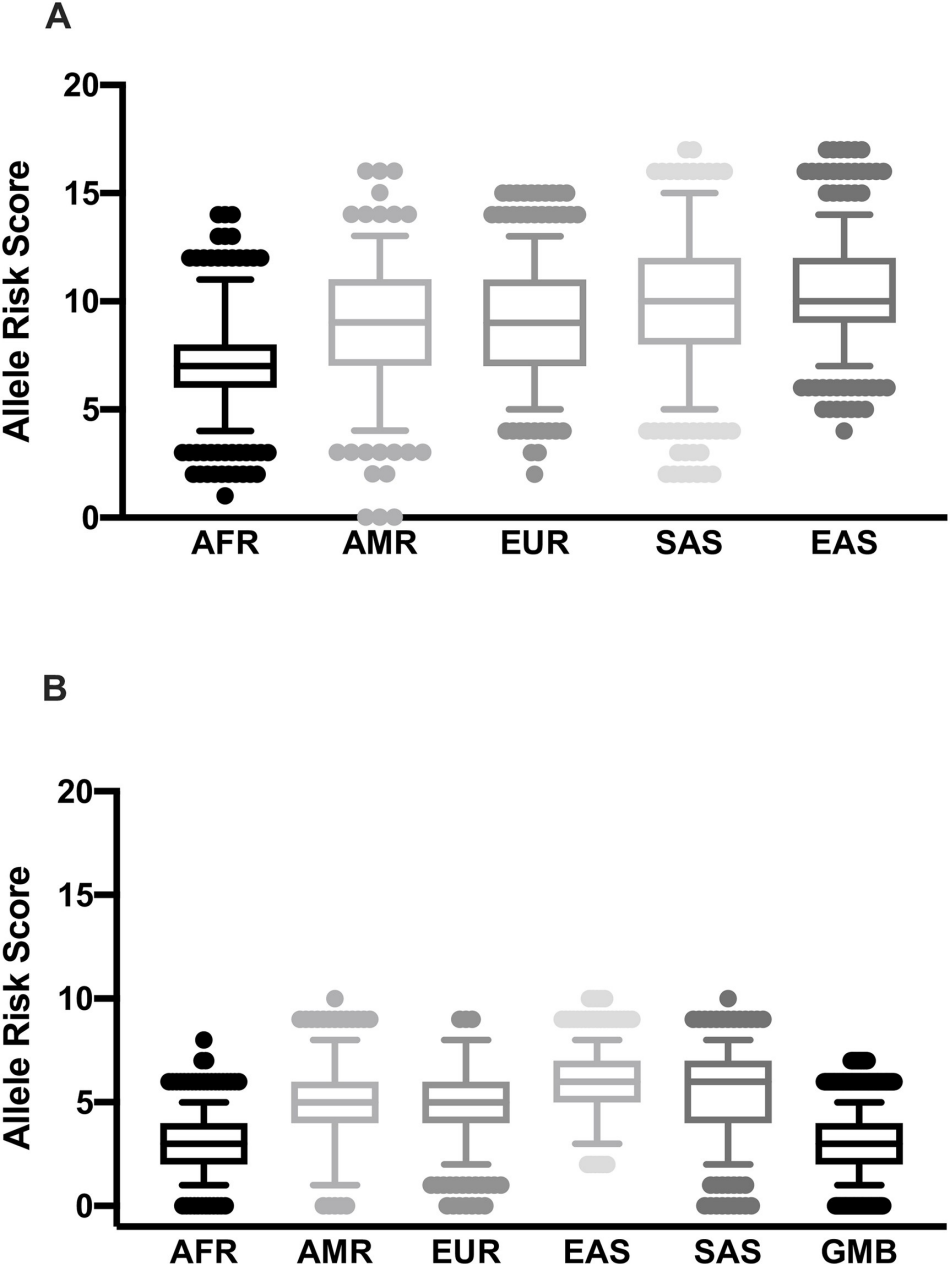
Distribution of the number of low iron risk alleles across global populations. (A) Distribution of the number of low iron risk alleles in eleven SNPs associated with low iron status across five populations. (B) Distribution of the number of low iron risk alleles in six SNPs with genotype data in the MRC Keneba Biobank population. Designation of the allele (risk or not) was determined by their previously published information as presented in S1 and S2 Tables. AFR, African; EUR, European; AMR, American; EAS, East Asian; SAS, South Asian.

The South and East Asian populations had the highest number of low iron risk alleles, whereas, Africans had the lowest and were significantly different from the other populations (Fig 6A, P < 0.0001). The American and European populations had similar number of low iron risk alleles, but lower than the Asian populations (P <0.0001) (Fig 6A).

Out of the eleven SNPs we found to be associated with low iron, it was only possible to compare six using the Keneba Biobank data, as data on the remaining SNPs were not available. The number of low iron risk alleles of the Gambians in the Keneba Biobank and the overall Africans in the 1000 Genomes were similar (Fig 6B). However, the low iron risk alleles in the Gambian and overall African populations were significantly lower compared to the other populations (P < 2x10^-16^) (Fig 6B).

**Fig 7:**
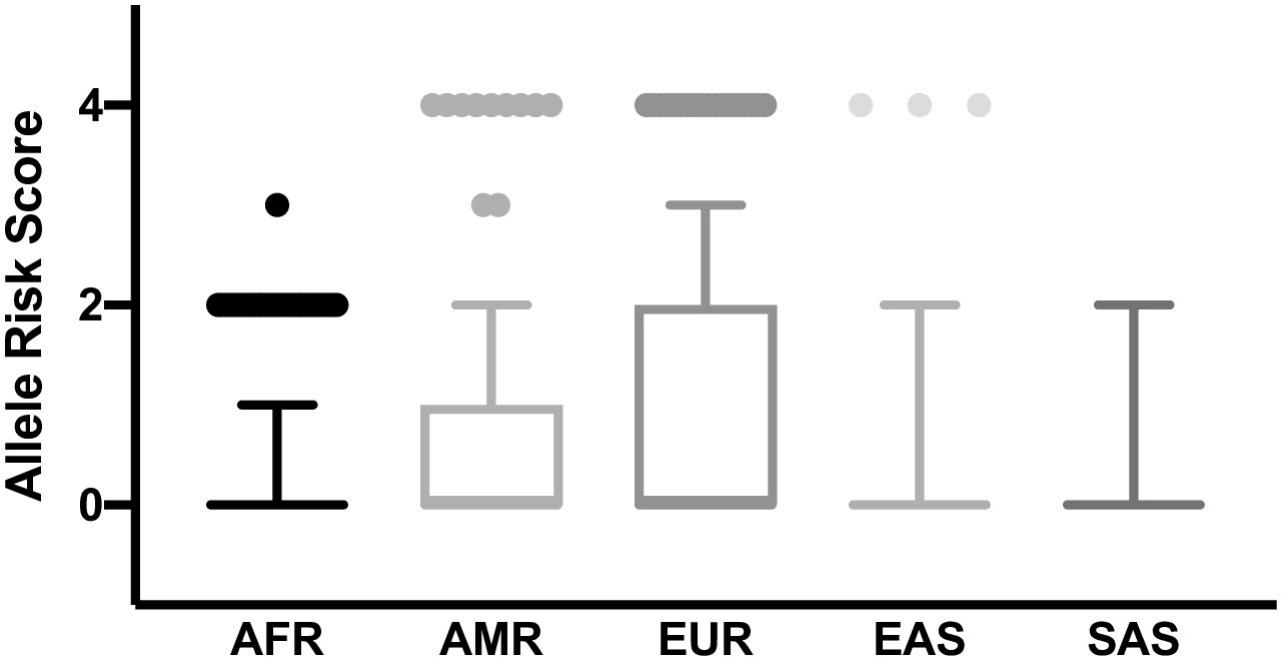
Distribution of the number of risk alleles for haemochromatosis among global populations. Designation of the risk allele was determined by previously published information as presented in S1 and S2 Tables. AFR, African; EUR, European; AMR, American; EAS, East Asian; SAS, South Asian.

Twelve SNPs were clearly associated with high iron or related biomarker (SNPs in *HAMP*, *TMPRSS6*, *TF*, *SLC40A1*, *TRF2* and close to *HFE*) with their risk alleles indicated (S1 Table). Three out of these twelve high iron associated SNPs were in or close to the *HFE* gene (rs1799945, rs1800562 and rs198846). These three SNPs were associated with haemochromatosis. Since haemochromatosis is predominantly common in those of European descent and rare in other populations, we analysed these SNPs separately. The European populations have the highest number of high iron risk alleles, significantly different from the other populations (P < 0.00850) (Fig 7).

**Fig 8:**
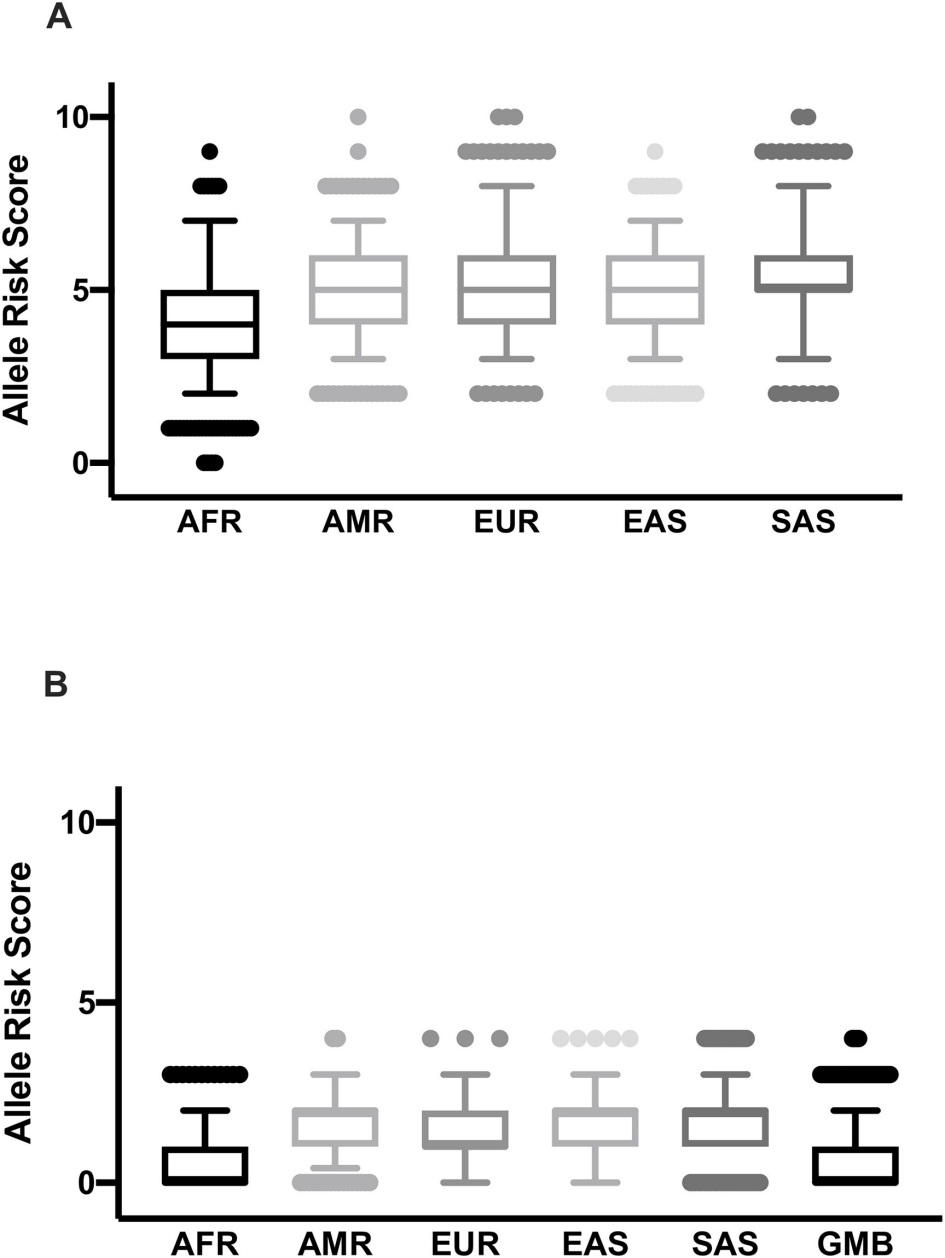
Distribution of the number of high iron risk alleles across global populations. (A) Distribution of the number of high iron risk alleles across five populations. (B) Distribution of the number of high iron risk alleles in three SNPs in the MRC Keneba Biobank (Gambian) and other populations. Designation of the allele (risk or not) was determined by their previously published information as presented in S1 and S2 Tables. AFR, African; EUR, European; AMR, American; EAS, East Asian; SAS, South Asian.

Data for two of the SNPs (rs1799945 and rs1800562) were available for the Keneba Biobank population, but the frequency of risk alleles was low (1% and 0%, respectively). Therefore, we could not compare the frequencies of risk alleles of these SNPs between the Keneba Biobank population and the 1000 Genomes project populations. Furthermore, we compared the frequencies of the high iron risk alleles of the remaining nine SNPs associated with elevated iron status in other genes. The African population in the 1000 Genomes Project had a significantly lower number of high iron risk alleles than the other populations (P <0.0001) (Fig 8A). The distributions between the other populations were similar. From these nine SNPs, genotype data for three SNPs (*TMPRSS6* rs5756506, *TF* rs1799852 and *SLC40A1* rs11568350 (Q248H) were available for the Gambians in the Keneba Biobank. When we compare the frequencies of the high iron risk alleles at these three SNPs across populations (Fig 8A), Gambians in the Keneba Biobank and pan-African populations have the lowest number of combined risk alleles for high iron (Fig 8B).

### Global population differentiation

We calculated the global and pairwise fixation index (*F*_*ST*_) across the 5 populations to assess population divergence for all iron-associated SNPs. The overall *F*_*ST*_ across the populations was 0.076. The pairwise *F*_*ST*_ between the continental groups shows that African versus non-African populations had the greatest allele frequency differentiation (*F*_ST_ >0.09; Table 1).

**Table 1:** Pairwise *F*_*ST*_ values between populations.

	EUR	EAS	AMR	SAS
EAS	0.0317			
AMR	0.0248	0.0232		
SAS	0.0263	0.0154	0.0130	
AFR	0.0992	0.1465	0.1507	0.1425

AFR, African; EUR, European; AMR, American; EAS, East Asian; SAS, South Asian; *F*_*ST*,_ fixation index.

**Fig 9:**
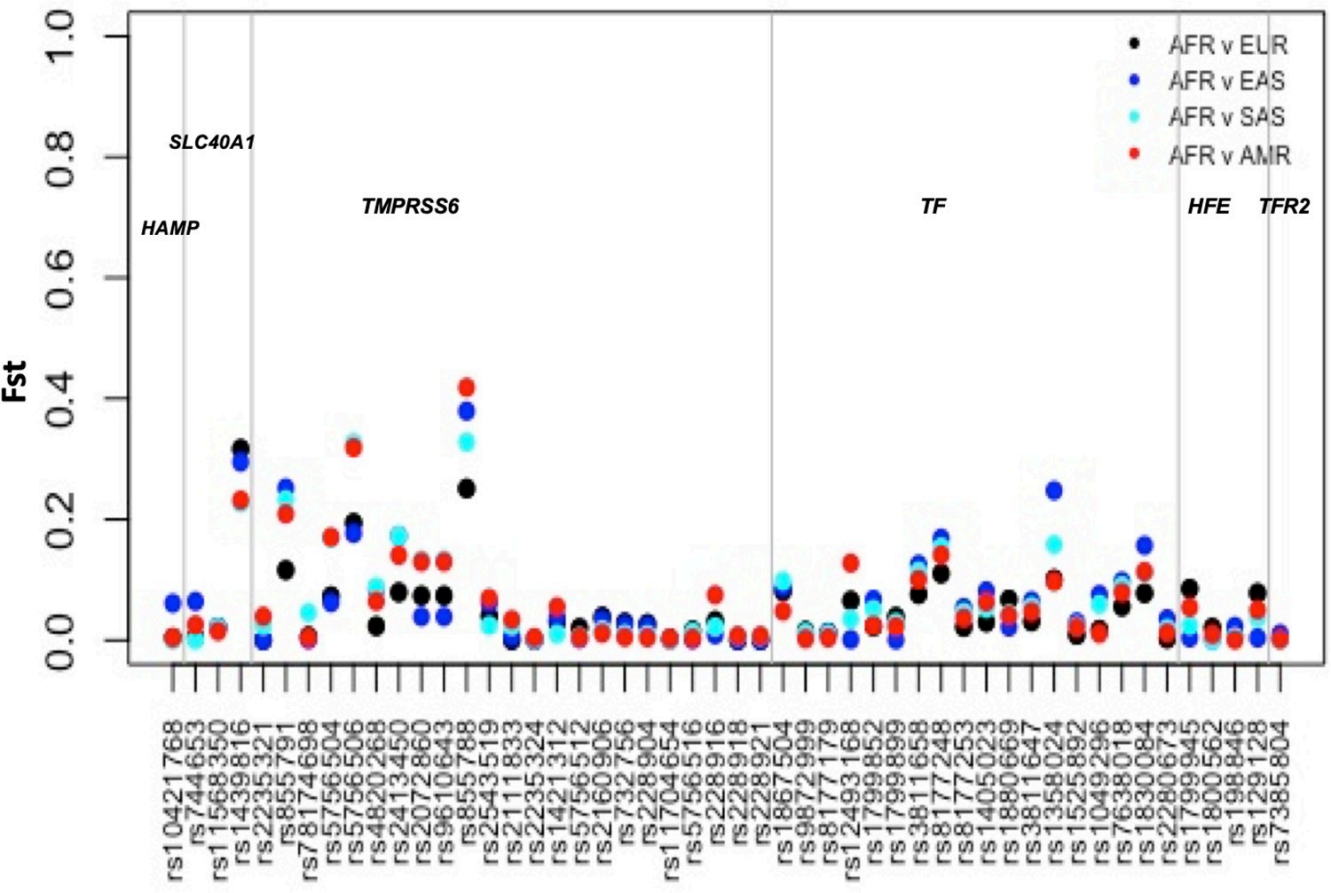
Pairwise F_ST_ values for iron related SNPs between African and non-African populations. This figure illustrates the comparison of *F*_*ST*_ scores between African and other global populations. AFR, African; AMR, American; EUR, European; EAS, East Asian; SAS, South Asian, *F*_*ST*_, fixation index.

We then investigated the individual SNPs driving the differentiation between African and other populations (Fig 9). The variants with the highest *F*_*ST*_ (>0.3) and highest allele frequency differences were rs1439816 in *SLC40A1* and rs855791, rs855788 and rs5756506 in *TMPRSS6* (Fig 9). The average *F*_*ST*_ values for the set of SNPs in each population was less than 0.065. The highest *F*_*ST*_ values we observed lay within the top 5% of the distribution of empirical global *F*_*ST*_ values described by others (95% percentile *F*_*ST*_ > 0.28) [43–45].

We also calculated the Population Branch Statistic (PBS) values, an *F*_*ST*_-based test involving the comparison of three populations, to investigate if the differentiation between populations could be driven by positive selection [40]. We used African, European and South Asian populations and observed that the PBS analysis reaffirms the *F*_*ST*_ results. In particular, the highest PBS values were present in: *SLC40A1* (rs1439816: AFR = 0.27, EUR = 0.05, SAS = 0.0) and *TMPRSS6* (rs855791: AFR = 0.16, EUR = 0.0, SAS = 0.07; rs855788: AFR = 0.29, EUR = 0, SAS = 0.04; rs5756506: AFR = 0.25, EUR = 0.0, SAS = 0.07) (S3 Table). These values are above the top five-percentile threshold of genome-wide PBS values (PBS> 0.156) described by others [41,42].

Finally, we investigated if any signals of recent positive selection could be detected in these genes by using the Integrated Haplotype Score (iHS) values from the Haplotter and HGDP selection browsers. The iHS statistic is based on the LD surrounding a positively selected allele compared with the LD around the alternative variant in the same position [46]. A positive iHS score (iHS > 2) means that the haplotypes on the ancestral allele background are longer than those with the derived allele [46]. A negative iHS score (iHS < -2) means that the haplotypes on the derived allele background are longer and are under selection. No clear evidence of selection was shown in the genomic regions containing *HAMP*, *TMPRSS6*, *TF and TRF2* (iHS<1). However, values of iHS scores close to 2 were found for the regions containing *SLC40A1* (e.g. rs1439816: iHS = 1.8 (East Asian-Hapmap ASN), iHS = 2 (European HapMap CEU) and *HFE* (e.g. rs198846: iHS = 1.8 ASN), suggesting a high frequency of longer haplotypes with the ancestral allele. Other studies have suggested that the *HEF* locus could be under positive selection in both European and Asian populations [75].

## Discussion

In this study we identified a significant lack of data on the genetic influences of iron status in African populations. This finding highlights a critical gap since African populations have high genetic diversity, and information from other populations may not be transferable to Africans [76,77]. African-specific studies on the genetic influences of iron status will help increase our understanding of the role played by genetic risk factors in the prevalence of anaemia in sub-Saharan Africa.

We used genotype data of populations from the Keneba Biobank at MRCG at LSHTM, The Gambia [33] and the 1000 Genome project [34] to describe the minor allele frequencies and differences in risk alleles in SNPs associated with iron imbalances or iron biomarkers. The allele frequencies of the available SNPs from the Gambian participants in the Keneba Biobank population were very similar to the Gambian population in the 1000 Genomes project. Both the Keneba Biobank population and 1000 Genomes Project included Gambians from the same ethnic group the Mandinka [33,34], which is the largest ethnic group in The Gambia. However, several other ethnic groups live in The Gambia, including Fula and Wolof ethic groups [78]. Variability in disease risk and nutrition status between the Fula and the Mandinka ethnic groups has been reported [79]. This finding is consistent with the inter-population genetic variability within African populations, which may also influence differences in disease susceptibility. Thus, future work could investigate the genetic diversity in the genes related to iron imbalances in non-Mandinka ethnic groups in The Gambia to determine their possible effect on impaired iron status.

Substantial differences in minor allele frequencies were observed when comparing the African versus non-African populations. The major differences occur in SNPs in *SLC40A1* and *TMPRSS6* genes. *SLC40A1* encodes ferroportin, a transmembrane transport protein which is the only known mammalian iron exporter [80]. The *SLC40A1* Q248H variant (rs11568350) is rare globally except in populations of African ancestry populations, where it reaches frequencies of ~5% [34]. The Q248H variant is associated with increased serum ferritin, decreased hepcidin concentrations and the risk of iron-loading in African populations [57,81]. Also, *SLC40A1* Q248H is associated with modest protection against anaemia and iron deficiency in African children [51,82].

We found significant differences in allelic frequencies for variants in the *TMPRSS6* gene which encodes for Matriptase-2, a type II transmembrane serine protease that negatively regulates hepcidin synthesis [23,83]. Impaired matriptase-2 activity leads to inappropriately raised hepcidin levels [84,85], which results in restricted iron absorption and release from storage sites [17]. Several SNPs in *TMPRSS6* had allele frequencies that are significantly different between African and non-African populations. These variants include rs855791, which has a low MAF (<10%) in African populations and reaches more than 35% in other populations. *TMPRSS6* rs855791 is associated with iron deficiency anaemia and IRIDA, with elevated hepcidin, reduced iron and reduced haemoglobin indices [20,21,84,86,87]. Differences in allele frequencies between continents have been described in many other genetic markers across the genome using data from the 1000 Genomes project [88,89] Therefore, the observed large allele frequency differences in SNPs associated with iron differences could be the result of demographic differences.

To understand if the differences in the observed allele frequencies could lead to differences in over- or under-representation of risk alleles leading to iron imbalances, we explored the frequencies of the combined risk alleles across the genes. We found that African populations, including the Gambian population from the Keneba Biobank, had a significantly lower number of alleles associated with the risk of anaemia or low iron. Similarly, we observed a lower number of risk alleles associated with high iron, or iron overload in Africans. This observation is likely because most of the studies were conducted in non-African populations. However, it is also possible that these differences are due to natural selection processes to balance the environmental risk factors to which African populations are exposed. For example, malnutrition and infections (e.g. helminths and malaria parasites) can lead to anaemia or limit iron overload which can increase susceptibility to certain infections (e.g bacterial). It is possible that the allele frequency differences between populations we described have occurred through founder effects as humans migrated out of Africa rather than through selective pressure. Possible signals of selection have only been observed for one SNP in *SLC40A1* and three SNPS in *TMPRSS6*, which have the highest *F*_*ST*_ and PBS values in Africa.

Our study has limitations. These include the potential for bias in the SNPs selection from the literature as there is an overrepresentation of studies related to genetics of iron imbalances in European and Asian populations. Also, it was difficult to ascertain the risk allele for several variants either because they were not described by the original study and/or the different studies used different genotyping platforms. In addition, although some risk alleles have been confirmed in more than one ethnic group (46% of the SNPs), for other SNPs it is possible that the alleles have different effects across populations and this could affect the risk allele analysis. Overall, our study highlights a major gap in genetic studies in Africa and the need to perform genetic studies in African populations.

We also observed a lower linkage disequilibrium between SNPs in African populations. For example, the *TMPRSS6* rs4820268 is in strong LD with *TMPRSS6* rs855791 in Europeans [90], but we found that these two SNPs are in weak LD in the Keneba Biobank population. This should be taken into account when performing association studies and selecting tag SNPs. In this setting, it may be easier to fine-scale map “causal” variants, but more difficult to identify the novel putative loci in a GWAS. Also, as iron imbalances can be due to multiple factors, it is critical to complement genetic studies with detailed meta-data collection, including detailed nutritional status, iron biomarkers, and clinical histories. Alternatively the effects of the variants can be studied prospectively using recall-by-genotype methods [91] that can also interrogate the dynamic responses to, for instance, the administration of iron supplements. Follow-up GWAS and candidate gene studies will be important to understand the genetic underpinning the geographic variation in the prevalence of iron imbalances disorders.

In conclusion, this study identified a substantial disparity in allele frequencies of genetic variants associated with iron, between Africans and other populations. We also, identified the scarcity of data on the genetic influences of iron status in Africa. Given the high burden of iron deficiency in sub-Saharan Africa, particularly in child-bearing women and children, comprehensive mapping of the genetic influences on iron status may help lay the foundation for future studies and assist in developing future iron intervention strategies.

## Supporting information

S1 Fig Linkage disequilibrium (LD) plots in SNPs in *HAMP*, *SLC40A1*, *TMPRSS6*, *TF*, *HFE* and *TFR2* genes. LD plot showing D prime values in SNPs associated with iron imbalances in (A) African populations, (B) European populations and (C) Gambian population in the Keneba Biobank.
(DOCX)

S1 Table Details of the fifty SNPs identified in the six genes that are associated with iron imbalance. (DOCX)

S2 Table Details of populations where each SNP was reported and the associated phenotypes. (DOCX)

S3 Table Population Branch Statistic (PBS) values involving the comparison of three populations. (DOCX)

S4 Table Genotyping data for 13 SNPs for the Gambian population in the Keneba Biobank. (XLSX)
