# Supplementary figures and images for: Differences in the frequency of genetic variants associated with iron imbalance among global populations
Source: PLoS One. 2020 Jul 1;15(7):e0235141. doi: 10.1371/journal.pone.0235141 (PMC7329092; doi:10.1371/journal.pone.0235141)

**A**


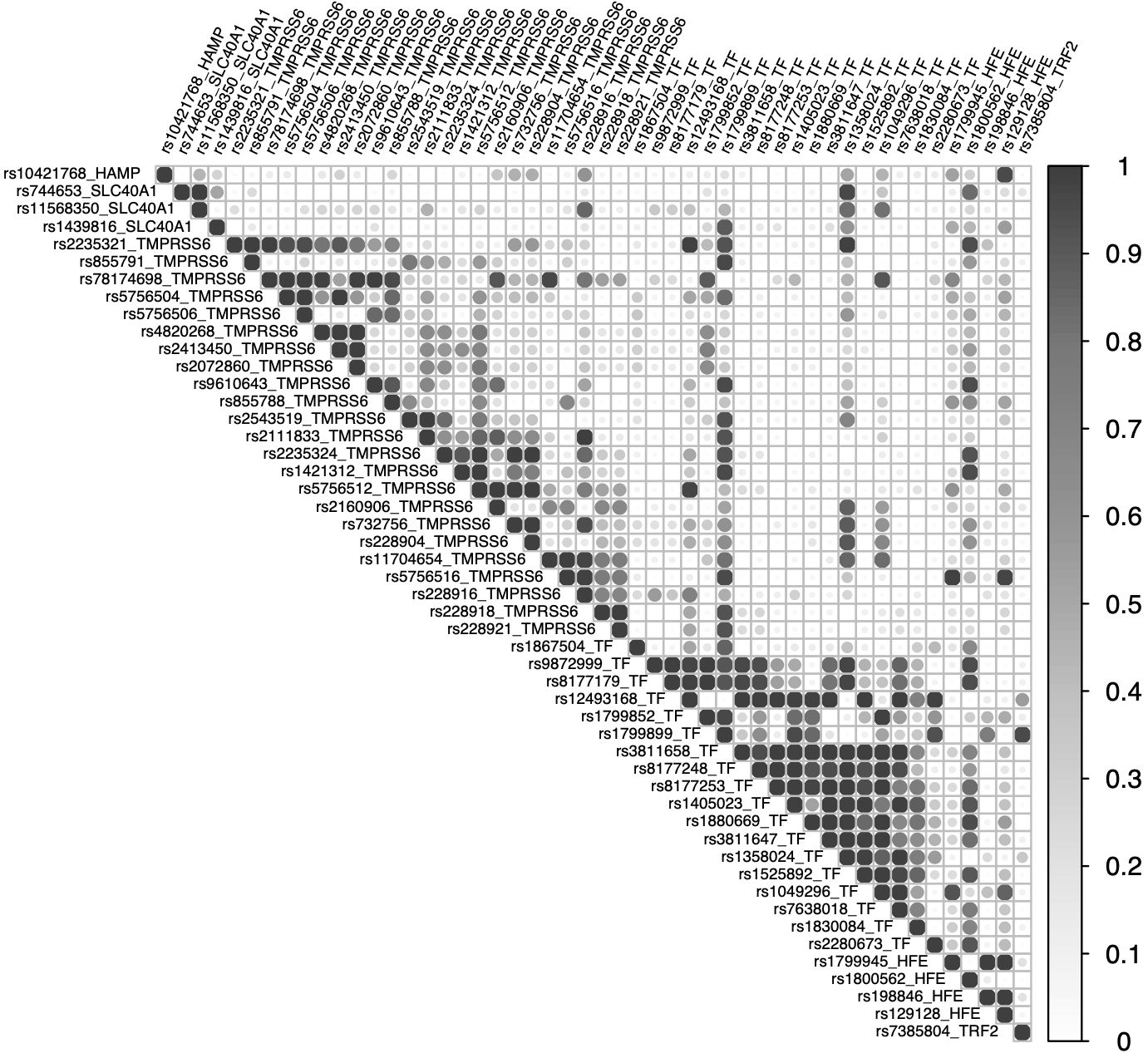


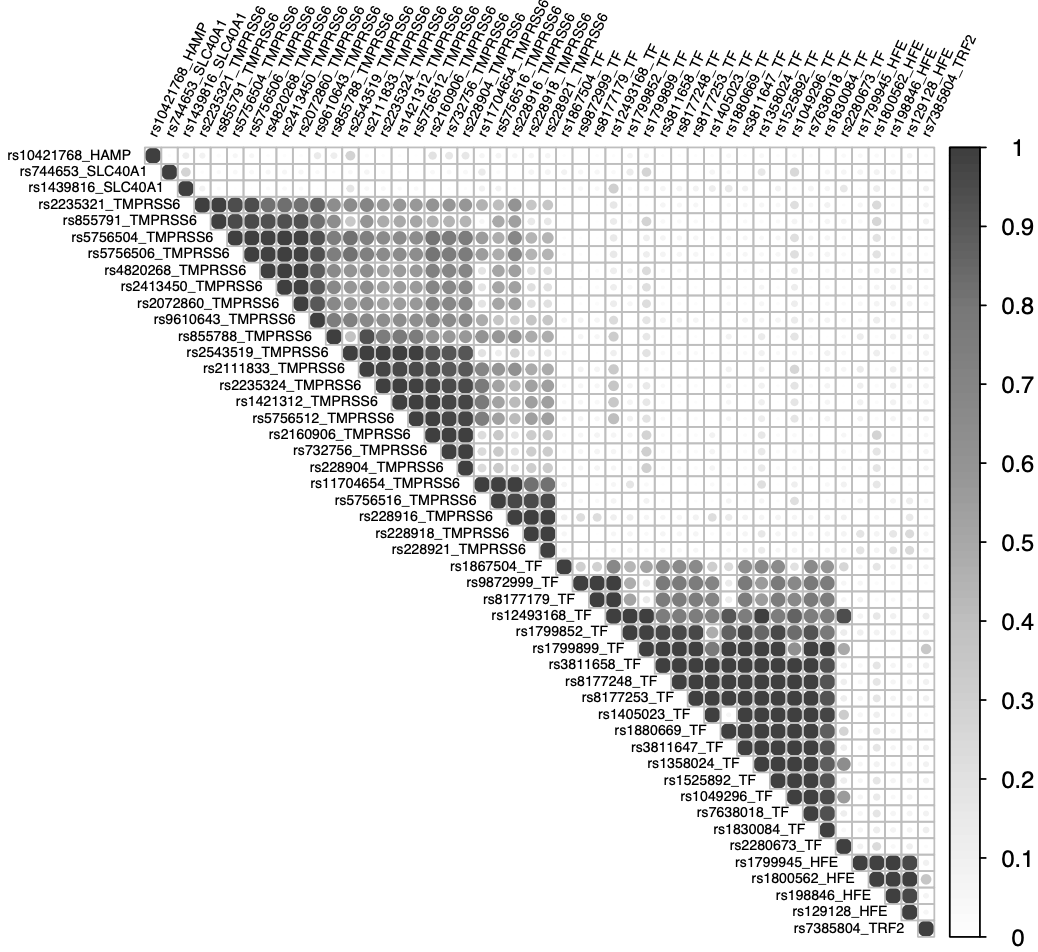


**B**


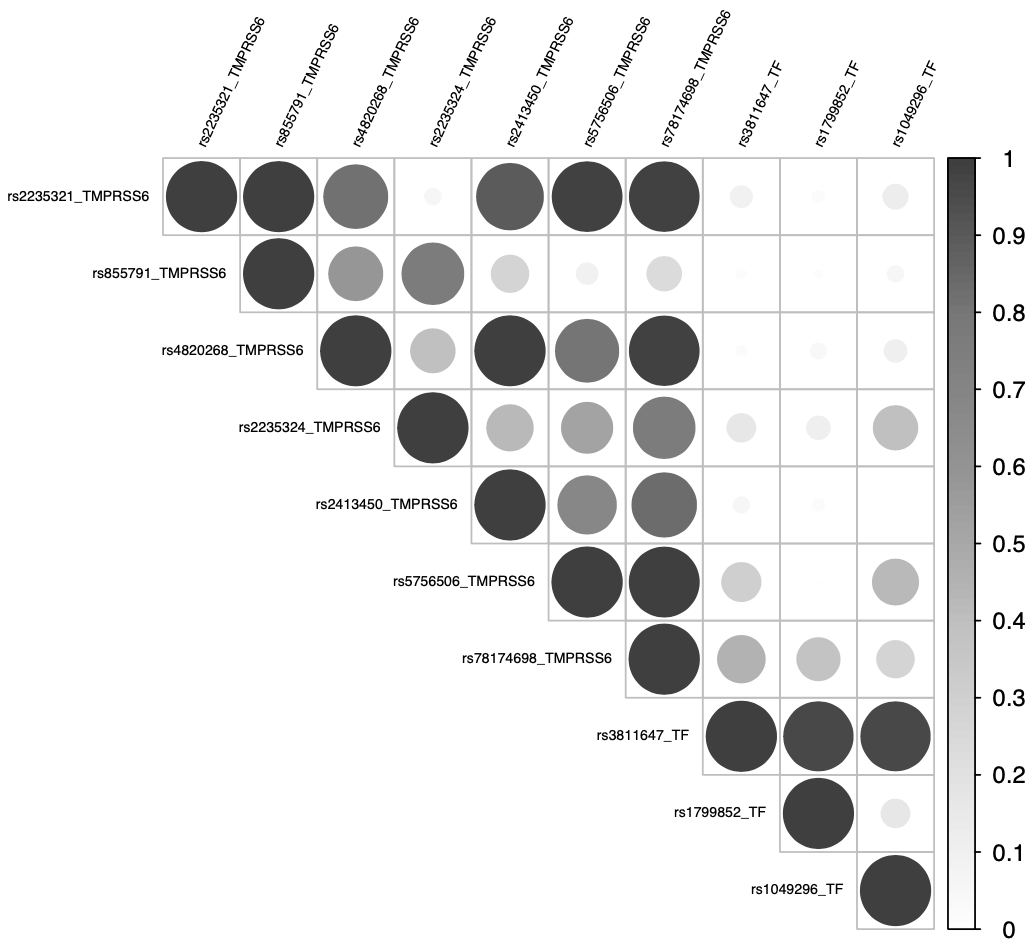


**C**

Supplement: S1 Fig — LD plot showing D prime values in SNPs associated with iron imbalances in (A) African populations, (B) European populations and (C) Gambian population in the Keneba Biobank. (DOCX) [file pone.0235141.s001.docx]
