# Supplementary material for: Differences in the frequency of genetic variants associated with iron imbalance among global populations
Source: PLoS One. 2020 Jul 1;15(7):e0235141. doi: 10.1371/journal.pone.0235141 (PMC7329092; doi:10.1371/journal.pone.0235141)
Supplement: S1 Table — (DOCX) [file pone.0235141.s002.docx]

| **SNPs** | **Loci** | **Type of variant (amino acid change)** | **Minor Allele** | **Major Allele** | **Risk allele** | **Effect on serum iron^1^** | **Minor Allele Frequency** | | | | | | | | | | **References** |
| --- | --- | --- | --- | --- | --- | --- | --- | --- | --- | --- | --- | --- | --- | --- | --- | --- | --- |
|  |  |  |  |  |  |  | 1000 Genomes | | | | | | | HapMap | | Keneba Biobank |  |
|  |  |  |  |  |  |  | Global (All) | AFR (All) | GWD | EUR | EAS | SAS | AMR | YRI | CEU |  |  |
| rs10421768 | *HAMP* | intron variant | G | A | A | High | 0.16 | 0.19 | 0.26 | 0.24 | 0.03 | 0.20 | 0.14 | 0.21 | 0.13 | NA | (1–4) |
| rs1799945 | *HFE* | missense variant (aa: H/D) | G | C | G | High | 0.07 | 0.01 | 0.00 | 0.17 | 0.03 | 0.07 | 0.12 | 0.01 | 0.13 | 0.01 | (5–17) |
| rs1800562 | *HFE* | Missense variant (C282Y) | A | G | A | High | 0.01 | 0.00 | 0.00 | 0.04 | 0.00 | 0.00 | 0.02 | 0.00 | 0.05 | 0 | (13,15,16,18–20) |
| rs198846 | *close to HFE* | Intron variant | A | G | A | High | 0.11 | 0.12 | 0.05 | 0.18 | 0.02 | 0.07 | 0.14 | 0.21 | 0.16 | NA | (21,22) |
| rs129128 | close to *HFE* | Intron variant | C | T | C | High | 0.07 | 0.01 | 0.00 | 0.16 | 0.03 | 0.09 | 0.11 | 0.01 | 0.14 | NA | (23) |
| rs744653 | close to *SLC40A1* | regulatory region variant | C | T | T | Moderates HH^2^ | 0.14 | 0.21 | 0.18 | 0.16 | 0.04 | 0.19 | 0.08 | 0.18 | 0.15 | NA | (5) |
| rs1439816 | *SLC40A1* | intron variant | C | G | G | Moderates HH^2^ | 0.34 | 0.73 | 0.74 | 0.16 | 0.18 | 0.25 | 0.23 | 0.76 | 0.17 | NA | (4) |
| rs11568350 (Q248H) | *SLC40A1* | Missense variant (Q248H) | A | C | A | High | 0.01 | 0.05 | 0.06 | 0.00 | 0.00 | 0.00 | 0.00 | 0.05 | 0.00 | 0.05 | (24–26) |
| rs2280673 | close to *TF* | intron variant RAB6B | A | C | NA | Low | 0.49 | 0.41 | 0.38 | 0.37 | 0.61 | 0.56 | 0.47 | 0.40 | 0.34 | NA | (18) |

**S1 Table:** **Details of the fifty SNPs identified in the six genes that are associated with** **iron imbalance**

| **Table S1 Continued**   \| **SNPs** \| **Loci** \| **Type of variant (amino acid change)** \| **Minor Allele** \| **Major Allele** \| **Risk allele** \| **Effect on serum iron^1^** \| **Minor Allele Frequency** \| \| \| \| \| \| \| \| \| \| **References** \| \| --- \| --- \| --- \| --- \| --- \| --- \| --- \| --- \| --- \| --- \| --- \| --- \| --- \| --- \| --- \| --- \| --- \| --- \| \|  \|  \|  \|  \|  \|  \|  \| 1000 Genomes \| \| \| \| \| \| \| HapMap \| \| Keneba Biobank \|  \| \|  \|  \|  \|  \|  \|  \|  \| Global  (All) \| AFR (All) \| GWD \| EUR \| EAS \| SAS \| AMR \| YRI \| CEU \|  \|  \| \| rs1867504 \| *TF* \| Intron variant \| A \| G \| A \| High \| 0.42 \| 0.22 \| 0.24 \| 0.49 \| 0.50 \| 0.52 \| 0.43 \| 0.48 \| 0.23 \| NA \| (2) \| \| rs9872999 \| *TF* \| Intron variant \| C \| T \| NA \| High \| 0.33 \| 0.35 \| 0.39 \| 0.47 \| 0.26 \| 0.25 \| 0.30 \| NA \| NA \| NA \| (12) \| \| rs8177179 \| *TF* \| Intron variant \| G \| A \| A \| Moderates HH^2^ \| 0.34 \| 0.36 \| 0.40 \| 0.47 \| 0.26 \| 0.26 \| 0.30 \| 0.28 \| 0.42 \| NA \| (5) \| \| rs1799852 \| *TF* \| Synonymous variant (L247L) \| A \| G \| A \| High \| 0.14 \| 0.05 \| 0.10 \| 0.14 \| 0.22 \| 0.19 \| 0.14 \| 0.06 \| 0.06 \| 0.07 \| (2,13,18,20,27) \| \| ﻿rs12493168 \| *TF* \|  \| G \| A \| NA \| Low \| 0.07 \| 0.01 \| 0.00 \| 0.13 \| 0.00 \| 0.08 \| 0.21 \| 0.01 \| 0.17 \| NA \| (27) \| \| rs1799899 (G277S) \| *TF* \| Missense variant (G277S) \| A \| G \| A \| Conflict^3^ \| 0.03 \| 0.00 \| 0.00 \| 0.07 \| 0.00 \| 0.05 \| 0.04 \| 0.00 \| 0.04 \| NA \| (28,29) \| \| rs3811658 \| *TF* \| Intron variant \| T \| C \| T \| Conflict^3^ \| 0.32 \| 0.12 \| 0.10 \| 0.35 \| 0.43 \| 0.41 \| 0.39 \| 0.01 \| 0.37 \| NA \| (2,27,30) \| \| rs8177248 \| *TF* \| intron variant \| T \| C \| NA \| Low \| 0.31 \| 0.08 \| 0.07 \| 0.35 \| 0.43 \| 0.41 \| 0.39 \| 0.04 \| 0.36 \| NA \| (30) \| \| rs8177253 \| *TF* \| intron variant \| T \| C \| T \| Low \| 0.35 \| 0.22 \| 0.15 \| 0.35 \| 0.43 \| 0.41 \| 0.40 \| 0.22 \| 0.36 \| NA \| (12) \| \| rs1405023 \| *TF* \| intron variant \| C \| T \| NA \| High \| 0.44 \| 0.62 \| 0.62 \| 0.44 \| 0.33 \| 0.38 \| 0.35 \| NA \| NA \| NA \| (27) \|   **Table S1 Continued**   \| **SNPs** \| **Loci** \| **Type of variant (amino acid change)** \| **Minor Allele** \| **Major Allele** \| **Risk allele** \| **Effect on serum iron^1^** \| **Minor Allele Frequency** \| \| \| \| \| \| \| \| \| \| **References** \| \| --- \| --- \| --- \| --- \| --- \| --- \| --- \| --- \| --- \| --- \| --- \| --- \| --- \| --- \| --- \| --- \| --- \| --- \| \|  \|  \|  \|  \|  \|  \|  \| 1000 Genomes \| \| \| \| \| \| \| HapMap \| \| Keneba Biobank \|  \| \|  \|  \|  \|  \|  \|  \|  \| Global (All) \| AFR (All) \| GWD \| EUR \| EAS \| SAS \| AMR \| YRI \| CEU \|  \|  \| \| rs1880669 \| *TF* \| intron variant \| T \| C \| NA \| Conflict^3^ \| 0.50 \| 0.65 \| 0.66 \| 0.39 \| 0.50 \| 0.44 \| 0.44 \| 0.70 \| 0.39 \| NA \| (27,30,31) \| \| rs3811647 \| *TF* \| intron variant \| A \| G \| A \| Low \| 0.34 \| 0.19 \| 0.15 \| 0.35 \| 0.42 \| 0.41 \| 0.39 \| 0.17 \| 0.36 \| 0.14 \| (6,12,13,18,27,32–36) \| \| rs1358024 \| *TF* \| intron variant \| T \| C \| NA \| Low \| 0.19 \| 0.01 \| 0.00 \| 0.19 \| 0.39 \| 0.27 \| 0.17 \| 0.00 \| 0.18 \| NA \| (18,27,30,33) \| \| rs1525892 \| *TF* \| intron variant \| A \| G \| A \| Conflict^3^ \| 0.36 \| 0.26 \| 0.23 \| 0.35 \| 0.47 \| 0.41 \| 0.40 \| 0.37 \| 0.23 \| NA \| (2,30,33) \| \| rs1049296 \| *TF* \| Missense variant (S589P) \| T \| C \| NA \| High \| 0.16 \| 0.06 \| 0.02 \| 0.14 \| 0.26 \| 0.23 \| 0.12 \| 0.07 \| 0.16 \| 0.01 \| (27) \| \| rs7638018 \| *TF* \| intron variant \| G \| A \| NA \| Low \| 0.33 \| 0.15 \| 0.14 \| 0.35 \| 0.42 \| 0.41 \| 0.40 \| 0.15 \| 0.36 \| NA \| (30) \| \| rs1830084 \| *TF* \| 3 prime UTR variant \| T \| A \| Uncertain risk allele^4^ \| Low \| 0.32 \| 0.11 \| 0.08 \| 0.34 \| 0.46 \| 0.40 \| 0.40 \| 0.13 \| 0.35 \| NA \| (12,30) \| \| rs7385804 \| *TFR2* \| Intron variant \| C \| A \| C \| Conflict^3^ \| 0.31 \| 0.33 \| 0.30 \| 0.38 \| 0.24 \| 0.32 \| 0.29 \| 0.35 \| 0.38 \| NA \| (2,5,6,31,32,37) \| \| rs2235321 \| *TMPRSS6* \| Synonymous variant (Y739Y) \| A \| G \| A \| Low \| 0.36 \| 0.41 \| 0.44 \| 0.42 \| 0.41 \| 0.26 \| 0.21 \| 0.38 \| 0.39 \| 0.44 \| (38–40) \| \| rs855791 \| *TMPRSS6* \| Missense variant (A736V) \| A \| G \| A \| Low \| 0.40 \| 0.10 \| 0.10 \| 0.39 \| 0.57 \| 0.54 \| 0.49 \| 0.12 \| 0.41 \| 0.07 \| (5,8,10,15–17,21,22,32,36,39,41–59) \| \| rs78174698 \| *TMPRSS6* \| missense variant (P555S) \| A \| G \| NA \| Low \| 0.03 \| 0.01 \| 0.02 \| 0.00 \| 0.01 \| 0.12 \| 0.00 \| 0.02 \| 0.00 \| 0.01 \| (43) \|   **Table S1 Continued**   \| **SNPs** \| **Loci** \| **Type of variant (amino acid change)** \| **Minor Allele** \| **Major Allele** \| **Risk allele** \| **Effect on serum iron^1^** \| **Minor Allele Frequency** \| \| \| \| \| \| \| \| \| \| **References** \| \| --- \| --- \| --- \| --- \| --- \| --- \| --- \| --- \| --- \| --- \| --- \| --- \| --- \| --- \| --- \| --- \| --- \| --- \| \|  \|  \|  \|  \|  \|  \|  \| 1000 Genomes \| \| \| \| \| \| \| HapMap \| \| Keneba Biobank \|  \| \|  \|  \|  \|  \|  \|  \|  \| Global (All) \| AFR (All) \| GWD \| EUR \| EAS \| SAS \| AMR \| YRI \| CEU \|  \|  \| \| rs5756504 \| *TMPRSS6* \| Intron variant \| T \| C \| T \| High \| 0.43 \| 0.67 \| 0.65 \| 0.40 \| 0.42 \| 0.26 \| 0.24 \| 0.71 \| 0.33 \| NA \| (22,56,57,60) \| \| rs5756506 \| *TMPRSS6* \| Intron variant \| C \| G \| C \| High \| 0.47 \| 0.83 \| 0.82 \| 0.40 \| 0.43 \| 0.26 \| 0.26 \| 0.85 \| 0.35 \| 0.84 \| (27,37,47) \| \| rs4820268 \| *TMPRSS6* \| Missense variant (D521E) \| G \| A \| G \| Low \| 0.46 \| 0.28 \| 0.27 \| 0.42 \| 0.56 \| 0.57 \| 0.53 \| 0.21 \| 0.48 \| 0.27 \| (2,6,21,22,32,39,42,47,57,61,62) \| \| rs2413450 \| *TMPRSS6* \| Intron variant \| T \| C \| T \| Low \| 0.42 \| 0.12 \| 0.12 \| 0.41 \| 0.56 \| 0.56 \| 0.52 \| 0.12 \| 0.48 \| 0.17 \| (2,47,53,63) \| \| rs2072860 \| *TMPRSS6* \| Intron variant \| G \| A \| NA \| Conflict^3^ \| 0.46 \| 0.28 \| 0.27 \| 0.42 \| 0.57 \| 0.57 \| 0.53 \| NA \| NA \| NA \| (12,43) \| \| rs9610643 \| *TMPRSS6* \| Intron variant \| A \| G \| NA \| Low \| 0.38 \| 0.60 \| 0.59 \| 0.33 \| 0.40 \| 0.23 \| 0.22 \| NA \| NA \| NA \| (43) \| \| rs855788 \| *TMPRSS6* \| intron variant \| A \| G \| NA \| High \| 0.49 \| 0.90 \| 0.86 \| 0.44 \| 0.30 \| 0.35 \| 0.27 \| 0.95 \| 0.31 \| NA \| (57) \| \| rs2543519 \| *TMPRSS6* \| Intron variant \| G \| A \| NA \| Low \| 0.25 \| 0.40 \| 0.43 \| 0.21 \| 0.17 \| 0.25 \| 0.14 \| 0.36 \| 0.21 \| NA \| (39,43) \| \| rs2111833 \| *TMPRSS6* \| Synonymous variant (S>S) \| T \| C \| T \| Conflict^3^ \| 0.31 \| 0.38 \| 0.31 \| 0.39 \| 0.31 \| 0.24 \| 0.20 \| 0.42 \| 0.34 \| NA \| (4) (30) \| \| rs2235324 \| *TMPRSS6* \| Missense variant (K253E) \| G \| A \| G \| Low \| 0.39 \| 0.40 \| 0.43 \| 0.43 \| 0.40 \| 0.37 \| 0.33 \| 0.43 \| 0.35 \| 0.45 \| (38–40,47,51,57) \|   **Table S1 Continued**   \| **SNPs** \| **Loci** \| **Type of variant (amino acid change)** \| **Minor Allele** \| **Major Allele** \| **Risk allele** \| **Effect on serum iron^1^** \| **Minor Allele Frequency** \| \| \| \| \| \| \| \| \| \| **References** \| \| --- \| --- \| --- \| --- \| --- \| --- \| --- \| --- \| --- \| --- \| --- \| --- \| --- \| --- \| --- \| --- \| --- \| --- \| \|  \|  \|  \|  \|  \|  \|  \| 1000 Genomes \| \| \| \| \| \| \| HapMap \| \| Keneba Biobank \|  \| \|  \|  \|  \|  \|  \|  \|  \| Global (All) \| AFR (All) \| GWD \| EUR \| EAS \| SAS \| AMR \| YRI \| CEU \|  \|  \| \| rs1421312 \| *TMPRSS6* \| intron variant \| G \| A \| NA \| High \| 0.47 \| 0.60 \| 0.58 \| 0.42 \| 0.40 \| 0.50 \| 0.35 \| 0.62 \| 0.47 \| NA \| (30,57) \| \| rs5756512 \| *TMPRSS6* \| intron variant \| T \| C \| NA \| Low \| 0.33 \| 0.28 \| 0.26 \| 0.42 \| 0.33 \| 0.36 \| 0.23 \| NA \| NA \| NA \| (43) \| \| rs2160906 \| *TMPRSS6* \| Intron variant \| A \| G \| NA \| Low \| 0.13 \| 0.06 \| 0.05 \| 0.19 \| 0.18 \| 0.14 \| 0.12 \| 0.05 \| 0.20 \| NA \| (36) \| \| rs732756 \| *TMPRSS6* \| Intron variant \| C \| T \| NA \| Low \| 0.14 \| 0.08 \| 0.08 \| 0.19 \| 0.18 \| 0.14 \| 0.12 \| 0.06 \| 0.20 \| NA \| (43) \| \| rs228904 \| *TMPRSS6* \| Intron variant \| G \| A \| NA \| High \| 0.14 \| 0.08 \| 0.08 \| 0.19 \| 0.18 \| 0.14 \| 0.12 \| 0.06 \| 0.20 \| NA \| (57) \| \| rs11704654 \| *TMPRSS6* \| Synonymous variant (P33P) \| T \| C \| NA \| Low \| 0.15 \| 0.15 \| 0.14 \| 0.19 \| 0.13 \| 0.16 \| 0.11 \| 0.16 \| 0.25 \| NA \| (39,42) \| \| rs5756516 \| *TMPRSS6* \| Intron variant \| T \| C \| NA \| Low \| 0.32 \| 0.30 \| 0.27 \| 0.42 \| 0.33 \| 0.20 \| 0.35 \| 0.31 \| 0.43 \| NA \| (43) \| \| rs228916 \| *TMPRSS6* \| 5 prime UTR variant \| C \| T \| T \| Low \| 0.07 \| 0.03 \| 0.00 \| 0.11 \| 0.00 \| 0.09 \| 0.18 \| 0.03 \| 0.08 \| NA \| (5) \| \| rs228921 \| *TMPRSS6* 2kb Upstream Variant \| Intergenic variant \| G \| A \| G \| Low \| 0.41 \| 0.40 \| 0.40 \| 0.41 \| 0.43 \| 0.48 \| 0.31 \| NA \| 0.40 \| NA \| (21,35) \| \| rs228918 \| *TMPRSS6:* 2kb Upstream \| Intergenic variant- \| A \| G \| G \| Low \| 0.41 \| 0.40 \| 0.40 \| 0.41 \| 0.43 \| 0.49 \| 0.31 \| 0.34 \| 0.47 \| NA \| (2,21) \| |
| --- | --- | --- | --- | --- | --- | --- | --- | --- | --- | --- | --- | --- | --- | --- | --- | --- | --- | --- | --- | --- | --- | --- | --- | --- | --- | --- | --- | --- | --- | --- | --- | --- | --- | --- | --- | --- | --- | --- | --- | --- | --- | --- | --- | --- | --- | --- | --- | --- | --- | --- | --- | --- | --- | --- | --- | --- | --- | --- | --- | --- | --- | --- | --- | --- | --- | --- | --- | --- | --- | --- | --- | --- | --- | --- | --- | --- | --- | --- | --- | --- | --- | --- | --- | --- | --- | --- | --- | --- | --- | --- | --- | --- | --- | --- | --- | --- | --- | --- | --- | --- | --- | --- | --- | --- | --- | --- | --- | --- | --- | --- | --- | --- | --- | --- | --- | --- | --- | --- | --- | --- | --- | --- | --- | --- | --- | --- | --- | --- | --- | --- | --- | --- | --- | --- | --- | --- | --- | --- | --- | --- | --- | --- | --- | --- | --- | --- | --- | --- | --- | --- | --- | --- | --- | --- | --- | --- | --- | --- | --- | --- | --- | --- | --- | --- | --- | --- | --- | --- | --- | --- | --- | --- | --- | --- | --- | --- | --- | --- | --- | --- | --- | --- | --- | --- | --- | --- | --- | --- | --- | --- | --- | --- | --- | --- | --- | --- | --- | --- | --- | --- | --- | --- | --- | --- | --- | --- | --- | --- | --- | --- | --- | --- | --- | --- | --- | --- | --- | --- | --- | --- | --- | --- | --- | --- | --- | --- | --- | --- | --- | --- | --- | --- | --- | --- | --- | --- | --- | --- | --- | --- | --- | --- | --- | --- | --- | --- | --- | --- | --- | --- | --- | --- | --- | --- | --- | --- | --- | --- | --- | --- | --- | --- | --- | --- | --- | --- | --- | --- | --- | --- | --- | --- | --- | --- | --- | --- | --- | --- | --- | --- | --- | --- | --- | --- | --- | --- | --- | --- | --- | --- | --- | --- | --- | --- | --- | --- | --- | --- | --- | --- | --- | --- | --- | --- | --- | --- | --- | --- | --- | --- | --- | --- | --- | --- | --- | --- | --- | --- | --- | --- | --- | --- | --- | --- | --- | --- | --- | --- | --- | --- | --- | --- | --- | --- | --- | --- | --- | --- | --- | --- | --- | --- | --- | --- | --- | --- | --- | --- | --- | --- | --- | --- | --- | --- | --- | --- | --- | --- | --- | --- | --- | --- | --- | --- | --- | --- | --- | --- | --- | --- | --- | --- | --- | --- | --- | --- | --- | --- | --- | --- | --- | --- | --- | --- | --- | --- | --- | --- | --- | --- | --- | --- | --- | --- | --- | --- | --- | --- | --- | --- | --- | --- | --- | --- | --- | --- | --- | --- | --- | --- | --- | --- | --- | --- | --- | --- | --- | --- | --- | --- | --- | --- | --- | --- | --- | --- | --- | --- | --- | --- | --- | --- | --- | --- | --- | --- | --- | --- | --- | --- | --- | --- | --- | --- | --- | --- | --- | --- | --- | --- | --- | --- | --- | --- | --- | --- | --- | --- | --- | --- | --- | --- | --- | --- | --- | --- | --- | --- | --- | --- | --- | --- | --- | --- | --- | --- | --- | --- | --- | --- | --- | --- | --- | --- | --- | --- | --- | --- | --- | --- | --- | --- | --- | --- | --- | --- | --- | --- | --- | --- | --- | --- | --- | --- | --- | --- | --- | --- | --- | --- | --- | --- | --- | --- | --- | --- | --- | --- | --- | --- | --- | --- | --- | --- | --- | --- | --- | --- | --- | --- | --- | --- | --- | --- | --- | --- | --- | --- | --- | --- | --- | --- | --- | --- | --- | --- | --- | --- | --- | --- | --- | --- | --- | --- | --- | --- | --- | --- | --- | --- | --- | --- | --- | --- | --- | --- | --- | --- | --- | --- | --- | --- | --- | --- | --- | --- | --- | --- | --- | --- | --- | --- | --- | --- | --- | --- | --- | --- | --- | --- | --- | --- | --- | --- | --- | --- | --- | --- | --- | --- | --- | --- | --- | --- | --- | --- | --- | --- | --- | --- | --- | --- | --- | --- | --- | --- | --- | --- | --- | --- | --- | --- | --- | --- | --- | --- | --- | --- | --- | --- | --- | --- | --- | --- | --- | --- | --- | --- | --- | --- | --- | --- | --- | --- | --- | --- | --- | --- | --- | --- | --- | --- | --- | --- | --- | --- | --- | --- | --- | --- | --- | --- | --- | --- | --- | --- | --- | --- | --- | --- | --- | --- | --- | --- | --- | --- | --- | --- | --- | --- | --- | --- | --- | --- | --- | --- | --- | --- | --- | --- | --- | --- | --- | --- | --- | --- | --- | --- | --- | --- | --- | --- | --- | --- | --- | --- | --- | --- | --- | --- | --- | --- | --- | --- | --- | --- | --- | --- | --- | --- | --- | --- | --- | --- | --- | --- | --- | --- | --- | --- | --- | --- | --- | --- | --- | --- | --- | --- | --- | --- | --- | --- | --- | --- | --- | --- | --- | --- | --- | --- | --- | --- | --- | --- | --- | --- | --- | --- | --- | --- | --- | --- | --- | --- | --- | --- | --- | --- | --- | --- | --- | --- | --- | --- | --- | --- | --- | --- | --- | --- | --- | --- | --- | --- | --- | --- | --- | --- | --- | --- | --- | --- | --- | --- | --- | --- | --- | --- | --- | --- | --- | --- | --- | --- | --- | --- | --- | --- | --- | --- | --- | --- | --- | --- | --- | --- | --- | --- | --- | --- | --- | --- | --- | --- | --- | --- | --- | --- | --- | --- | --- | --- | --- | --- | --- | --- | --- | --- | --- | --- | --- | --- | --- | --- | --- | --- | --- | --- | --- | --- | --- | --- | --- | --- | --- | --- | --- | --- | --- | --- | --- | --- | --- | --- | --- | --- | --- | --- | --- | --- | --- | --- | --- | --- | --- | --- | --- | --- | --- | --- | --- | --- | --- | --- | --- | --- | --- | --- | --- | --- | --- | --- | --- | --- | --- | --- | --- | --- | --- | --- | --- | --- | --- | --- | --- | --- | --- | --- | --- | --- | --- | --- | --- | --- | --- | --- | --- | --- | --- | --- | --- | --- | --- | --- | --- | --- | --- | --- | --- | --- | --- | --- | --- | --- | --- | --- | --- | --- | --- | --- | --- | --- | --- | --- | --- | --- | --- | --- | --- | --- | --- | --- | --- | --- |

^1^ The documented effect of each SNP on iron status, based on its influence on iron biomarkers. High: Indicates SNPs that have been associated with elevated iron status as shown by at least iron biomarker signifying elevated iron status. Low indicates SNPs associated with decreased iron status, determined by at least one biomarker signifying low iron.

^2^ The only information available about this SNPs is that they modulate hemochromatosis.

^3^ We found contradictory information about the effects of these SNPs on iron status. Different papers reported direction of effects of these SNPs on iron status.

NA indicates SNPs that we could not establish the risk allele because it was not stated by the respective studies that reported the SNPs. In the Keneba Biobank, NA indicates SNPs whose genotype data was not present in the Biobank population.

^4^Indicates a SNP in which the effect of the risk allele has not been clearly described in the paper it was reported.

^5^The phenotype associated with the risk allele

AFR, Africans; AMR, Americans; CEU, Utah residents with Northern and Western European ancestry from the CEPH collection; EAS, East Asians; EUR, Europeans; GWD, Gambians from Western Division; HAMP, hepcidin antimicrobial peptide; Hb, haemoglobin; HCT, haematocrit; HFE, High fe; HH, hereditary hemochromatosis; IDA, iron deficiency anaemia; MCH, mean corpuscular haemoglobin; NA, not available; SAS, South Asians; SI, serum iron; *SLC40A1*, solute carrier family 40 member 1; SNP, single nucleotide polymorphism; sTfR, soluble transferrin receptor; *TF*, transferrin; *TMPRSS6*, transmembrane protease serine 6; UTR, untranslated region; YRI, Yoruba in Nigeria.

|  |  |  |  |
| --- | --- | --- | --- |

**Reference:**

1. Andreani M, Radio FC, Testi M, De Bernardo C, Troiano M, Majore S, et al. Association of hepcidin promoter c.-582 A>G variant and iron overload in thalassemia major. Haematologica. 2009;94(9):1293–6.

2. Gichohi-Wainaina WN, Tanaka T, Towers GW, Verhoef H, Veenemans J, Talsma EF, et al. Associations between Common Variants in Iron-Related Genes with Haematological Traits in Populations of African Ancestry. PLoS One [Internet]. 2016;11(6):e0157996. Available from: http://www.ncbi.nlm.nih.gov/pubmed/27332551

3. Javaheri-Kermani M, Farazmandfar T, Ajami A, Yazdani Y. Impact of hepcidin antimicrobial peptide on iron overload in tuberculosis patients. Scand J Infect Dis [Internet]. 2014;46(10):693–6. Available from: http://informahealthcare.com/doi/abs/10.3109/00365548.2014.929736

4. Radio FC, Majore S, Aurizi C, Sorge F, Biolcati G, Bernabini S, et al. Hereditary hemochromatosis type 1 phenotype modifiers in Italian patients. The controversial role of variants in HAMP, BMP2, FTL and SLC40A1 genes. Blood Cells Mol Dis [Internet]. 2015 Jun;55(1):71–5. Available from: http://dx.doi.org/10.1016/j.bcmd.2015.04.001

5. Benyamin B, Esko T, Ried JS, Radhakrishnan A, Vermeulen SH, Traglia M, et al. Novel loci affecting iron homeostasis and their effects in individuals at risk for hemochromatosis. Nat Commun [Internet]. 2014 Oct 29;5(2):4926. Available from: http://www.ncbi.nlm.nih.gov/pubmed/25352340

6. Pichler I, Minelli C, Sanna S, Tanaka T, Schwienbacher C, Naitza S, et al. Identification of a common variant in the TFR2 gene implicated in the physiological regulation of serum iron levels. Hum Mol Genet [Internet]. 2011 Mar 15;20(6):1232–40. Available from: http://www.ncbi.nlm.nih.gov/pubmed/21208937

7. Garewal G, Das R, Ahluwalia J, Marwaha RK. Prevalence of the H63D mutation of the HFE in north India: Its presence does not cause iron overload in beta thalassemia trait. Eur J Haematol. 2005;74(4):333–6.

8. Sørensen E, Rigas AS, Thørner LW, Burgdorf KS, Pedersen OB, Petersen MS, et al. Genetic factors influencing ferritin levels in 14,126 blood donors: Results from the Danish Blood Donor Study. Transfusion. 2016;56(3):622–7.

9. Mast AE, Lee T-H, Schlumpf KS, Wright DJ, Johnson B, Carrick DM, et al. The impact of HFE mutations on haemoglobin and iron status in individuals experiencing repeated iron loss through blood donation*. Br J Haematol [Internet]. 2012 Feb;156(3):388–401. Available from: http://www.ncbi.nlm.nih.gov/pubmed/22118647

10. Athiyarath R, Shaktivel K, Abraham V, Singh D, Bondu JD, Chapla A, et al. Association of genetic variants with response to iron supplements in pregnancy. Genes Nutr [Internet]. 2015 Jul 30;10(4):25. Available from: http://link.springer.com/10.1007/s12263-015-0474-2

11. Whitfield JB, Cullen LM, Jazwinska EC, Powell LW, Heath AC, Zhu G, et al. Effects of HFE C282Y and H63D polymorphisms and polygenic background on iron stores in a large community sample of twins. Am J Hum Genet [Internet]. 2000 Apr;66(4):1246–58. Available from: http://www.ncbi.nlm.nih.gov/pubmed/10739755

12. Li J, Lange LA, Duan Q, Lu Y, Singleton AB, Zonderman AB, et al. Genome-wide admixture and association study of serum iron, ferritin, transferrin saturation and total iron binding capacity in African Americans. Hum Mol Genet. 2015;24(2):572–81.

13. Blanco-Rojo R, Baeza-Richer C, López-Parra AM, Pérez-Granados AM, Brichs A, Bertoncini S, et al. Four variants in transferrin and HFE genes as potential markers of iron deficiency anaemia risk: an association study in menstruating women. Nutr Metab (Lond) [Internet]. 2011 Oct 6;8:69. Available from: http://www.ncbi.nlm.nih.gov/pubmed/21978626

14. Beutler E, Felitti V, Gelbart T, Waalen J. Haematological effects of the C282Y HFE mutation in homozygous and heterozygous states among subjects of northern and southern European ancestry. Br J Haematol. 2003;120(5):887–93.

15. Galesloot TE, Geurts-Moespot AJ, den Heijer M, Sweep FCGJ, Fleming RE, Kiemeney L a LM, et al. Associations of common variants in HFE and TMPRSS6 with iron parameters are independent of serum hepcidin in a general population: a replication study. J Med Genet [Internet]. 2013;50(9):593–8. Available from: http://www.ncbi.nlm.nih.gov/pubmed/23794717

16. De Falco L, Tortora R, Imperatore N, Bruno M, Capasso M, Girelli D, et al. The role of TMPRSS6 and HFE variants in iron deficiency anemia in celiac disease. Am J Hematol. 2018;93(3):383–93.

17. Pichler I, Del Greco M F, Gögele M, Lill CM, Bertram L, Do CB, et al. Serum iron levels and the risk of Parkinson disease: a Mendelian randomization study. PLoS Med [Internet]. 2013;10(6):e1001462. Available from: http://www.ncbi.nlm.nih.gov/pubmed/23750121

18. Benyamin B, McRae AF, Zhu G, Gordon S, Henders AK, Palotie A, et al. Variants in TF and HFE explain approximately 40% of genetic variation in serum-transferrin levels. Am J Hum Genet [Internet]. 2009 Jan;84(1):60–5. Available from: http://dx.doi.org/10.1016/j.ajhg.2008.11.011

19. Gordeuk VR, Brannon PM. Ethnic and genetic factors of iron status in women of reproductive age. Am J Clin Nutr. 2017;106:1594S-1599S.

20. Blanco-Rojo R, Toxqui L, López-Parra AM, Baeza-Richer C, Pérez-Granados AM, Arroyo-Pardo E, et al. Influence of diet, menstruation and genetic factors on iron status: A cross-sectional study in Spanish women of childbearing age. Int J Mol Sci. 2014;15(3):4077–87.

21. Chambers JC, Zhang W, Li Y, Sehmi J, Wass MN, Zabaneh D, et al. Genome-wide association study identifies variants in TMPRSS6 associated with hemoglobin levels. Nat Genet [Internet]. 2009 Nov;41(11):1170–2. Available from: http://www.ncbi.nlm.nih.gov/pubmed/19820698

22. Kullo IJ, Ding K, Jouni H, Smith CY, Chute CG. A Genome-Wide Association Study of Red Blood Cell Traits Using the Electronic Medical Record. 2010;5(9):1–9.

23. Chen Z, Tang H, Qayyum R, Schick UM, Nalls MA, Handsaker R, et al. Genome-wide association analysis of red blood cell traits in African Americans: The cogent network. Hum Mol Genet. 2013;22(12):2529–38.

24. Kasvosve I, Gomo ZAR, Nathoo KJ, Matibe P, Mudenge B, Loyevsky M, et al. Effect of ferroportin Q248H polymorphism on iron status in African children. 2018;(April):1102–6.

25. Masaisa F, Breman C, Gahutu JB, Mukiibi J, Delanghe J, Philippé J. Ferroportin (SLC40A1) Q248H mutation is associated with lower circulating serum hepcidin levels in Rwandese HIV-positive women. Ann Hematol. 2012;91(6):911–6.

26. Rivers CA, Barton JC, Gordeuk VR, Acton RT, Speechley MR, Snively BM, et al. Association of ferroportin Q248H polymorphism with elevated levels of serum ferritin in African Americans in the Hemochromatosis and Iron Overload Screening (HEIRS) Study. Blood Cells, Mol Dis. 2007;38(3):247–52.

27. Constantine CC, Anderson GJ, Vulpe CD, Mclaren CE, Bahlo M, Yeap HL, et al. A novel association between a SNP in CYBRD1 and serum ferritin levels in a cohort study of HFE hereditary haemochromatosis. 2009;(August):140–9.

28. Sarria B, Lopez-parra AM, Perez-granados AM, Arroyo-pardo E, Roe MA, Teucher B, et al. The G277S transferrin mutation does not affect iron absorption in iron deficient women. 2007;57–60.

29. Lee PL, Halloran C, Trevino R, Felitti V, Beutler E. Human transferrin G277S mutation: a risk factor for iron deficiency anaemia. Br J Haematol [Internet]. 2001 Nov;115(2):329–33. Available from: http://www.ncbi.nlm.nih.gov/pubmed/11703331

30. McLaren CE, McLachlan S, Garner CP, Vulpe CD, Gordeuk VR, Eckfeldt JH, et al. Associations between single nucleotide polymorphisms in iron-related genes and iron status in multiethnic populations. PLoS One. 2012;7(6).

31. Piao W, Wang L, Zhang T, Wang Z, Shangguan S, Sun J, et al. A single-nucleotide polymorphism in transferrin is associated with soluble transferrin receptor in Chinese adolescents. Asia Pac J Clin Nutr. 2017;26(6):1170–8.

32. An P, Wu Q, Wang H, Guan Y, Mu M, Liao Y, et al. TMPRSS6, but not TF, TFR2 or BMP2 variants are associated with increased risk of iron-deficiency anemia. Hum Mol Genet. 2012;21(9):2124–31.

33. McLaren CE, Garner CP, Constantine CC, McLachlan S, Vulpe CD, Snively BM, et al. Genome-wide association study identifies genetic loci associated with iron deficiency. PLoS One. 2011;6(3).

34. Koller DL, Imel EA, Lai D, Padgett LR, Acton D, Gray A, et al. Genome-wide association study of serum iron phenotypes in premenopausal women of European descent. Blood Cells, Mol Dis [Internet]. 2016;57:50–3. Available from: http://dx.doi.org/10.1016/j.bcmd.2015.12.002

35. Gichohi-Wainaina, W. N. Melse-Boonstra, A. Swinkels, D. W. Zimmermann, M. B. Feskens, E. J. Towers GW. Common variants and haplotypes in the TF, TNF- alpha , and TMPRSS6 genes are associated with iron status in a female black South. J Nutr 2015. 2015;145(5):945–53.

36. Benyamin B, Ferreira MAR, Willemsen G, Gordon S, Middelberg RPS, McEvoy BP, et al. Common variants in TMPRSS6 are associated with iron status and erythrocyte volume. Nat Genet [Internet]. 2009 Nov 11;41(11):1173–5. Available from: http://www.nature.com/doifinder/10.1038/ng.456

37. Soranzo N, Spector TD, Mangino M, Kühnel B, Rendon A, Teumer A, et al. A genome-wide meta-analysis identifies 22 loci associated with eight hematological parameters in the HaemGen consortium. Nat Genet [Internet]. 2009;41(11):1182–90. Available from: http://dx.doi.org/10.1038/ng.467

38. Lee PL, Barton JC, Khaw PL, Bhattacharjee SY, Barton JC. Common TMPRSS6 mutations and iron, erythrocyte, and pica phenotypes in 48 women with iron deficiency or depletion. Blood Cells, Mol Dis [Internet]. 2012 Feb;48(2):124–7. Available from: http://dx.doi.org/10.1016/j.bcmd.2011.12.003

39. Delbini P, Vaja V, Graziadei G, Duca L, Nava I, Refaldi C, et al. Genetic variability of TMPRSS6 and its association with iron deficiency anaemia. Br J Haematol. 2010;151(3):281–4.

40. Poggiali E, Andreozzi F, Nava I, Consonni D, Graziadei G, Cappellini MD. The role of TMPRSS6 polymorphisms in iron deficiency anemia partially responsive to oral iron treatment. Am J Hematol. 2015;90(4):306–9.

41. Galesloot TE, Verweij N, Traglia M, Barbieri C, Van Dijk F, Geurts-Moespot AJ, et al. Meta-GWAS and meta-analysis of exome array studies do not reveal genetic determinants of serum hepcidin. PLoS One. 2016;11(11):1–13.

42. Kloss-Brandstätter A, Erhart G, Lamina C, Meister B, Haun M, Coassin S, et al. Candidate gene sequencing of SLC11A2 and TMPRSS6 in a family with severe anaemia: Common SNPs, rare haplotypes, no causative mutation. PLoS One. 2012;7(4):1–8.

43. Bhatia P, Singh A, Hegde A, Jain R, Bansal D. Systematic evaluation of paediatric cohort with iron refractory iron deficiency anaemia (IRIDA) phenotype reveals multiple TMPRSS6 gene variations. Br J Haematol [Internet]. 2017 Apr;177(2):311–8. Available from: http://doi.wiley.com/10.1111/bjh.14554

44. Valenti L, Fracanzani AL, Rametta R, Fraquelli M, Soverini G, Pelusi S, et al. Effect of the A736V TMPRSS6 polymorphism on the penetrance and clinical expression of hereditary hemochromatosis. J Hepatol [Internet]. 2012;57(6):1319–25. Available from: http://dx.doi.org/10.1016/j.jhep.2012.07.041

45. Traglia M, Girelli D, Biino G, Campostrini N, Corbella M, Sala C, et al. Association of HFE and TMPRSS6 genetic variants with iron and erythrocyte parameters is only in part dependent on serum hepcidin concentrations. J Med Genet [Internet]. 2011 Sep;48(9):629–34. Available from: http://www.ncbi.nlm.nih.gov/pubmed/21785125

46. Gan W, Guan Y, Wu Q, An P, Zhu J, Lu L, et al. Association of TMPRSS6 polymorphisms with ferritin, hemoglobin, and type 2 diabetes risk in a Chinese Han population. Am J Clin Nutr [Internet]. 2012 Mar;95(3):626–32. Available from: http://www.ncbi.nlm.nih.gov/pubmed/22301935

47. Batar B, Bavunoglu I, Hacioglu Y, Cengiz M, Mutlu T, Yavuzer S, et al. The role of TMPRSS6 gene variants in iron-related hematological parameters in Turkish patients with iron deficiency anemia. Gene [Internet]. 2018;673(January):201–5. Available from: https://doi.org/10.1016/j.gene.2018.06.055

48. Nai A, Pagani A, Silvestri L, Campostrini N, Corbella M, Girelli D, et al. TMPRSS6 rs855791 modulates hepcidin transcription in vitro and serum hepcidin levels in normal individuals. Blood [Internet]. 2011 Oct 20;118(16):4459–62. Available from: http://www.bloodjournal.org/cgi/doi/10.1182/blood-2011-06-364034

49. Danquah I, Gahutu J-B, Zeile I, Musemakweri A, Mockenhaupt FP. Anaemia, iron deficiency and a common polymorphism of iron-regulation, TMPRSS6 rs855791, in Rwandan children. Trop Med Int Health [Internet]. 2014;19(1):117–22. Available from: http://www.ncbi.nlm.nih.gov/pubmed/24175968

50. Pei SN, Ma MC, You HL, Fu HC, Kuo CY, Rau KM, et al. TMPRSS6 rs855791 polymorphism influences the susceptibility to iron deficiency anemia in women at reproductive age. Int J Med Sci. 2014;11(6):614–9.

51. Beutler E, Van Geet C, te Loo DMWM, Gelbart T, Crain K, Truksa J, et al. Polymorphisms and mutations of human TMPRSS6 in iron deficiency anemia. Blood Cells, Mol Dis [Internet]. 2010 Jan 15;44(1):16–21. Available from: http://www.ncbi.nlm.nih.gov/pubmed/19818657

52. Pelusi S, Girelli D, Rametta R, Campostrini N, Alfieri C, Traglia M, et al. The A736V TMPRSS6 polymorphism influences hepcidin and iron metabolism in chronic hemodialysis patients: TMPRSS6 and hepcidin in hemodialysis. BMC Nephrol [Internet]. 2013;14:48. Available from: http://www.pubmedcentral.nih.gov/articlerender.fcgi?artid=3585892&tool=pmcentrez&rendertype=abstract

53. Ganesh SK, Zakai NA, van Rooij FJA, Soranzo N, Smith A V, Nalls MA, et al. Multiple loci influence erythrocyte phenotypes in the CHARGE Consortium. Nat Genet [Internet]. 2009 Nov 11;41(11):1191–8. Available from: http://dx.doi.org/10.1038/ng.466

54. van der Harst P, Zhang W, Mateo Leach I, Rendon A, Verweij N, Sehmi J, et al. Seventy-five genetic loci influencing the human red blood cell. Nature. 2013;492(7429):369–75.

55. Bédard A, Lewis SJ, Burgess S, John Henderson A, Shaheen SO. Maternal iron status during pregnancy and respiratory and atopic outcomes in the offspring: A Mendelian randomisation study. BMJ Open Respir Res. 2018;5(1):1–10.

56. Kamatani Y, Matsuda K, Okada Y, Kubo M, Hosono N, Daigo Y, et al. Genome-wide association study of hematological and biochemical traits in a Japanese population. Nat Genet [Internet]. 2010;42(3):210–5. Available from: http://dx.doi.org/10.1038/ng.531

57. Tanaka T, Roy CN, Yao W, Matteini A, Semba RD, Arking D, et al. A genome-wide association analysis of serum iron concentrations. Blood [Internet]. 2010 Jan 7;115(1):94–6. Available from: http://www.ncbi.nlm.nih.gov/pubmed/19880490

58. Valenti L, Rametta R, Dongiovanni P, Motta BM, Canavesi E, Pelusi S, et al. The A736V TMPRSS6 Polymorphism Influences Hepatic Iron Overload in Nonalcoholic Fatty Liver Disease. PLoS One. 2012;7(11).

59. Cheng HL, Hancock DP, Rooney KB, Steinbeck KS, Grif HJ, Connor HTO. SHORT COMMUNICATION A candidate gene approach for identifying differential iron responses in young overweight women to an energy-restricted haem iron-rich diet. 2014;(February):1250–2.

60. Seiki T, Naito M, Hishida A, Takagi S, Matsunaga T, Sasakabe T, et al. Association of genetic polymorphisms with erythrocyte traits: Verification of SNPs reported in a previous GWAS in a Japanese population. Gene [Internet]. 2018;642(October 2017):172–7. Available from: http://dx.doi.org/10.1016/j.gene.2017.11.031

61. Alfred T, Ben-Shlomo Y, Cooper R, Hardy R, Deary IJ, Elliott J, et al. Genetic variants influencing biomarkers of nutrition are not associated with cognitive capability in middle-aged and older adults. J Nutr [Internet]. 2013 May;143(5):606–12. Available from: http://jn.nutrition.org/cgi/doi/10.3945/jn.112.171520

62. Ji Y, Flower R, Hyland C, Saiepour N, Faddy H. Genetic factors associated with iron storage in Australian blood donors. Blood Transfus. 2018;16(2):123–9.

63. Guo MH, Nandakumar SK, Ulirsch JC, Zekavat SM, Buenrostro JD, Natarajan P, et al. Comprehensive population-based genome sequencing provides insight into hematopoietic regulatory mechanisms. Proc Natl Acad Sci [Internet]. 2017;114(3):E327–36. Available from: http://www.pnas.org/lookup/doi/10.1073/pnas.1619052114

64. Jackson HA, Carter K, Darke C, Guttridge MG, Ravine D, Hutton RD, et al. HFE mutations, iron deficiency and overload in 10 500 blood donors. Br J Haematol. 2001;114(2):474–84.
