# Supplementary material for: Differences in the frequency of genetic variants associated with iron imbalance among global populations
Source: PLoS One. 2020 Jul 1;15(7):e0235141. doi: 10.1371/journal.pone.0235141 (PMC7329092; doi:10.1371/journal.pone.0235141)
Supplement: S2 Table — (DOCX) [file pone.0235141.s003.docx]

| **S2 Table**  **Details of populations where each SNP was reported and the associated phenotypes**   \| **SNPs** \| **Gene** \| **Minor allele** \| **Major allele** \| **Risk Allele** \| **Study/ first author** \| **Location/ Population/ Ethnicity^1^** \| **Associated trait^2^** \| **Reference^3^** \| \| --- \| --- \| --- \| --- \| --- \| --- \| --- \| --- \| --- \| \| rs10421768 \| *HAMP* \| G \| A \| A \| Javaheri-Kermani et al., 2014 \| Iran \| Associated with elevated serum iron and low hepcidin levels \| (3) \| \| rs10421768 \| *HAMP* \| G \| A \| A \| Andreani et al., 2009 \| Italy \| Associated with elevated liver iron concentration and raised serum ferritin levels \| (1) \| \| rs10421768 \| *HAMP* \| G \| A \| A \| Radio et al., 2016 \| Italy \| Homozygotes and heterozygotes has signicficantly reduced transferrin levels \| (4) \| \| rs10421768 \| *HAMP* \| G \| A \| A \| Gichohi-Wainaina et al., 2016 \| Kenyans, Tanzanians, S. Africans and African-Americans \| Significant increased in Hb in Kenyans only \| (2) \| \| rs1049296 \| *TF* \| T \| C \| NA \| ﻿Constantine et al., 2009 \| European ancestry \| Reduced TSAT and serum transferrin \| (27) \| \| rs11568350 (Q248H) \| *SLC40A1* \| A \| C \| A \| Masaisa et al., 2012 \| Rwanda \| Associated with low hepcidin and transferrin, and elevated serum ferritin \| (25) \| \| rs11568350 (Q248H) \| *SLC40A1* \| A \| C \| A \| Kasvosve et al., 2018 \| Zimbabwe \| Associated with elevated ferritin levels and protection against IDA \| (24) \| \| rs11568350 (Q248H) \| *SLC40A1* \| A \| C \| A \| Rivers et al., 2007 \| African-Americans \| Associated with elevated serum ferritin in men \| (26) \| \| rs11704654 \| *TMPRSS6* \| T \| C \| NA \| Delbini et al., 2010 \| Italy \| Associated with iron deficiency \| (39) \| \| rs11704654 \| *TMPRSS6* \| T \| C \| NA \| Kloss-Brandstatter et al., 2012 \| Netherlands \| Associated with increased serum iron and ferritin \| (42) \| \| rs12493168 \| *TF* \| G \| A \| NA \| Constantine et al., 2009 \| European ancestry \| Elevated serum transferritin \| (27) \|  \| **SNPs** \| **Gene** \| **Minor allele** \| **Major allele** \| **Risk Allele** \| **Study/ first author** \| **Location/ Population/ Ethnicity^1^** \| **Associated trait^2^** \| **Reference^3^** \| \| --- \| --- \| --- \| --- \| --- \| --- \| --- \| --- \| --- \| \| rs129128 \| *HFE* \| C \| T \| C \| Li et al., 2015 \| USA \| Elevated serum iron \| (12) \| \| rs1358024 \| *TF* \| T \| C \| NA \| Constantine et al., 2009 \| European ancestry \| Elevated serum transferritin \| (27) \| \| rs1358024 \| *TF* \| T \| C \| NA \| Benyamin et al., 2009 \| Aurtralians \| Affects serum transferrin concentrations, but direction of effect was not stated \| (18) \| \| rs1405023 \| *TF* \| C \| T \| NA \| Constantine et al., 2009 \| European ancestry \| Reduced serum transferrin levels \| (27) \| \| rs1421312 \| *TMPRSS6* \| G \| A \| NA \| McLaren et al., 2012 \| Whites, African-Americans, Hispanics and Asians (US & Canada) \| Increased serum iron and TSAT, and decreased sTfR in Whites \| (30) \| \| rs1421312 \| *TMPRSS6* \| G \| A \| NA \| Tanaka et al., 2010 \| Italy and USA \| Significantly associated with reduced iron status \| (57) \| \| rs1439816 \| *SLC40A1* \| C \| G \| G \| Radio et al., 2016 \| Italy \| Moderates hereditary hemochromatosis \| (4) \| \| rs1525892 \| *TF* \| A \| G \| A \| Gichohi-Wainaina et al., 2016 \| Kenyans, Tanzanians, S. Africans and African-Americans \| Marginally significant higher ferritin concentrations \| (2) \| \| rs1525892 \| *TF* \| A \| G \| A \| McLaren et al., 2012 \| Whites, African-Americans, Hispanics and Asians (US & Canada) \| Elevated TIBC in all the populations \| (30) \| \| rs1799852 \| *TF* \| A \| G \| A \| Gichohi-Wainaina et al., 2016 \| South Africa \| The A allele is associated with lower serum ferritin \| (2) \|   **Table S3 continued**   \| **SNPs** \| **Gene** \| **Minor allele** \| **Major allele** \| **Risk Allele** \| **Study/ first author** \| **Location/ Population/ Ethnicity^1^** \| **Associated trait^2^** \| **Reference^3^** \| \| --- \| --- \| --- \| --- \| --- \| --- \| --- \| --- \| --- \| \| rs1799852 \| *TF* \| A \| G \| A \| Benyamin, et al., 2009 \| Australian of European ancestry \| Decreased transferrin, increased serum iron, ferritin and TSAT \| (18) \| \| rs1799852 \| *TF* \| A \| G \| A \| Blanco-Rojo et al., 2011 \| Spain \| Reduced serum transferrin levels \| (13) \| \| rs1799852 \| *TF* \| A \| G \| A \| Constantine et al., 2009 \| European ancestry \| Reduced serum transferrin levels \| (27) \| \| rs1799899 (G277S) \| *TF* \| A \| G \| A \| Sarria et al., 2007 \| Spain \| No significant differences in iron biomarkers between genotypes \| (28) \| \| rs1799899 (G277S) \| *HFE* \| A \| G \| A \| Lee et al., 2001 \| European ancestry \| The variant allele predispose to iron deficiency \| (29) \| \| rs1799945 (H63D) \| *HFE* \| G \| C \| G \| Pichler et al., 2011 \| Italy and USA \| Reduced serum iron \| (6) \| \| rs1799945 (H63D) \| *HFE* \| G \| C \| G \| Athiyarath et al., 2015 \| India \| The G allele was significantly associated with adequate response to iron supplementation \| (10) \| \| rs1799945 (H63D) \| *HFE* \| G \| C \| G \| Blanco-Rojo et al., 2011 \| Spain \| Reduced serum transferrin levels \| (13) \| \| rs1799945 (H63D) \| *HFE* \| G \| C \| G \| Galesloot et al., 2013 \| Netherlands \| Significantly associated with iron and TSAT \| (15) \| \| rs1799945 (H63D) \| *HFE* \| G \| C \| G \| De Falco et al., 2018 \| Italy \| associated with elevated Hb, MCV, serum iron and ferritin levels \| (16) \| \| rs1799945 (H63D) \| *HFE* \| G \| C \| G \| Mast et al., 2012 \| Multicenter: USA \| Increased iron stores \| (9) \| \| rs1799945 (H63D) \| *HFE* \| G \| C \| G \| Sørensen et al., 2015 \| Denmark \| The C alleles are associated with iron deficiency in women \| (8) \|   **Table S3 continued**   \| **SNPs** \| **Gene** \| **Minor allele** \| **Major allele** \| **Risk Allele** \| **Study/ first author** \| **Location/ Population/ Ethnicity^1^** \| **Associated trait^2^** \| **Reference^3^** \| \| --- \| --- \| --- \| --- \| --- \| --- \| --- \| --- \| --- \| \| rs1799945 (H63D) \| *HFE* \| G \| C \| G \| Whitfield et al., 2000 \| Australia \| Associated with high iron stores \| (11) \| \| rs1799945 (H63D) \| *HFE* \| G \| C \| G \| Benyamin et al., 2014 \| European ancestry \| Reduced serum iron, TSAT and ferritin, and elevated transferrin \| (5) \| \| rs1799945 (H63D) \| *HFE* \| G \| C \| G \| Li et al., 2015 \| USA \| Elevated serum iron \| (12) \| \| rs1799945 (H63D) \| *HFE* \| G \| C \| G \| Pichler et al., 2013 \| European Ancestry populations \| Increased iron stores \| (17) \| \| rs1799945 (H63D) \| *HFE* \| G \| C \| G \| Blanco-Rojo et al., 2014 \| European ancestry \| Associated with protection against iron deficiency \| (20) \| \| rs1799945 (H63D) \| *HFE* \| G \| C \| G \| Garewal et al., 2005 \| India \| No effect on iron status \| (7) \| \| rs1799945 (H63D) \| *HFE* \| G \| C \| G \| Beutler et al., 2003 \| European ancestry \| Elevated Hb, TSAT, ferritin and lower anaemia prevalence \| (14) \| \| rs1799945 (H63D) \| *HFE* \| G \| C \| G \| Jackson et al., 2001 \| UK: Wales \| Elevated serum ferritin, TSAT and Hb, and reduced UIBC \| (64) \| \| rs1800562 (C282Y) \| *HFE* \| A \| G \| A \| Benyamin, et al., 2009 \| Australian of European ancestry \| Increased serum iron, ferritin and TSAT, decreased transferrin \| (18) \| \| rs1800562 (C282Y) \| *HFE* \| A \| G \| A \| Kullo et al., 2010 \| USA \| Elevated MCH \| (22) \| \| rs1800562 (C282Y) \| *HFE* \| A \| G \| A \| Blanco-Rojo et al., 2011 \| Spain \| Reduced serum transferrin levels \| (13) \| \| rs1800562 (C282Y) \| *HFE* \| A \| G \| A \| Galesloot et al., 2013 \| Netherlands \| Significantly associated with ferritin, iron, TSAT and TIBC \| (15) \| \| rs1800562 (C282Y) \| *HFE* \| A \| G \| A \| Benyamin et al., 2009 \| Aurtralians \| Reduced transferrin, raised serum iron, TSAT, ferritin, Hb and MCV \| (36) \|   **Table S3 continued**   \| **SNPs** \| **Gene** \| **Minor allele** \| **Major allele** \| **Risk Allele** \| **Study/ first author** \| **Location/ Population/ Ethnicity^1^** \| **Associated trait^2^** \| **Reference^3^** \| \| --- \| --- \| --- \| --- \| --- \| --- \| --- \| --- \| --- \| \| rs1800562 (C282Y) \| *HFE* \| A \| G \| A \| Seiki et al., 2018 \| Japanese \| Elevated MCV, Hb \| (60) \| \| rs1800562 (C282Y) \| *HFE* \| A \| G \| A \| Gordeuk et al., 2017 \| Multi-ethnic: USA and Canada \| Associated with elevated ferritin levels \| (19) \| \| rs1800562 (C282Y) \| *HFE* \| A \| G \| A \| De Falco et al., 2018 \| Italians \| Carriage of C282Y was higher in celiac disease cases in than in controls. \| (16) \| \| rs1800562 (C282Y) \| *HFE* \| A \| G \| A \| Sørensen et al., 2015 \| Denmark \| The G allele is associated with lower iron stores \| (8) \| \| rs1800562 (C282Y) \| *HFE* \| A \| G \| A \| Whitfield et al., 2000 \| Australians \| Associated with high iron stores \| (11) \| \| rs1800562 (C282Y) \| *HFE* \| A \| G \| A \| Benyamin et al., 2014 \| European ancestry \| Elevated iron, TSAT and ferritin, and reduced tranferrin \| (5) \| \| rs1800562 (C282Y) \| *HFE* \| A \| G \| A \| Li et al., 2015 \| USA \| Elevated ferritin and low TIBC \| (12) \| \| rs1800562 (C282Y) \| *HFE* \| A \| G \| A \| Pichler et al., 2013 \| European Ancestry population \| Increased iron stores \| (17) \| \| rs1800562 (C282Y) \| *HFE* \| A \| G \| A \| Traglia et al., 2011 \| Italians \| Elevated ferritin and decreased hepcidi/ferritin ration \| (45) \| \| rs1800562 (C282Y) \| *HFE* \| A \| G \| A \| Koller et al., 2016 \| European-American (USA) \| Associated with TIBC \| (34) \| \| rs1800562 (C282Y) \| *HFE* \| A \| G \| G \| Bedard et al., 2018 \| UK \| G alleles associated with reduced iron stores \| (55) \| \| rs1800562 (C282Y) \| *HFE* \| A \| G \| A \| McLaren et al., 2011 \| GWAS on Americans of European ancestry \| Decreased TIBC and UIBC \| (33) \| \| rs1800562 (C282Y) \| *HFE* \| A \| G \| A \| Beutler et al., 2003 \| European ancestry \| Elevated Hb, TSAT, ferritin and lower anaemia prevalence \| (14) \|   **Table S3 continued**   \| **SNPs** \| **Gene** \| **Minor allele** \| **Major allele** \| **Risk Allele** \| **Study/ first author** \| **Location/ Population/ Ethnicity^1^** \| **Associated trait^2^** \| **Reference^3^** \| \| --- \| --- \| --- \| --- \| --- \| --- \| --- \| --- \| --- \| \| rs1800562 (C282Y) \| *HFE* \| A \| G \| A \| Jackson et al., 2001 \| UK: Wales \| Elevated serum ferritin, TSAT and Hb, and reduced UIBC; compound heterozygotes of H63D and C282Y has has high iron stores \| (64) \| \| rs1830084 \| *TF* \| T \| A \| A \| Benyamin, et al., 2009 \| Australian of European ancestry \| increased transferrin \| (18) \| \| rs1830084 \| *TF* \| T \| A \| T \| Li et al., 2015 \| USA \| Elevated TIBC \| (12) \| \| rs1867504 \| *TF* \| A \| G \| A \| Gichohi-Wainaina et al., 2016 \| Kenyans, Tanzanians, S. Africans and African-Americans \| Elevated ferritin levels \| (2) \| \| rs1880669 \| *TF* \| T \| C \| NA \| McLaren et al., 2012 \| Whites, African-Americans, Hispanics and Asians (US & Canada) \| Elevated TIBC in all the populations \| (30) \| \| rs1880669 \| *TF* \| T \| C \| NA \| ﻿Constantine et al., 2009 \| European ancestry \| Reduced serum transferrin levels \| (27) \| \| rs1880669 \| *TF* \| T \| C \| A \| Piao et al., 2017 \| Chinese adolescents \| A allele is associated with highet sTfR \| (31) \| \| rs198846 \| *HFE* \| A \| G \| A \| Kullo et al., 2010 \| USA \| Elevated MCV and MCH \| (22) \| \| rs198846 \| *HFE* \| A \| G \| A \| Chambers et al., 2009 \| European and Indian Ancestry \| The major allele (G) are associated with lower Hb concentration \| (21) \| \| rs2072860 \| *TMPRSS6* \| G \| A \| NA \| Bhathia et al., 2017 \| India \| Associated with IRIDA \| (43) \| \| rs2072860 \| *TMPRSS6* \| G \| A \| A \| Li et al., 2015 \| USA \| Elevated serum iron \| (12) \| \| rs2111833 \| *TMPRSS6* \| T \| C \| T \| McLaren et al., 2012 \| Whites, African-Americans, Hispanics and Asians (US & Canada) \| Increased serum iron and TSAT in Whites \| (30) \|   **Table S3 Continued**   \| **SNPs** \| **Gene** \| **Minor allele** \| **Major allele** \| **Risk Allele** \| **Study/ first author** \| **Location/ Population/ Ethnicity^1^** \| **Associated trait^2^** \| **Reference^3^** \| \| --- \| --- \| --- \| --- \| --- \| --- \| --- \| --- \| --- \| \| rs2111833 \| *TMPRSS6* \| T \| C \| T \| Radio et al., 2016 \| Italy \| No significant differences in iron biomarkers between genotypes \| (4) \| \| rs2160906 \| *TMPRSS6* \| A \| G \| NA \| Tanaka et al., 2010 \| Italy and USA \| Significantly associated with reduced iron status \| (57) \| \| rs2235321 \| *TMPRSS6* \| A \| G \| A \| Lee et al., 2012 \| White Americans \| Decreased TSAT \| (38) \| \| rs2235321 \| *TMPRSS6* \| A \| G \| A \| Delbini et al., 2010 \| Italy \| Associated with iron deficiency \| (39) \| \| rs2235321 \| *TMPRSS6* \| A \| G \| A \| Poggiali et al., 2015 \| Italy \| Associated with iron deficiecy \| (40) \| \| rs2235324 \| *TMPRSS6* \| G \| A \| G \| Lee et al., 2012 \| White Americans \| Elevated TSAT \| (38) \| \| rs2235324 \| *TMPRSS6* \| G \| A \| G \| Beutler et al., 2010 \| Caucasians \| Associated with iron deficiency \| (51) \| \| rs2235324 \| *TMPRSS6* \| G \| A \| NA \| Delbini et al., 2010 \| Italy \| Associated with iron deficiency \| (39) \| \| rs2235324 \| *TMPRSS6* \| G \| A \| NA \| Tanaka et al., 2010 \| Italy and USA \| Significantly associated with reduced iron status \| (57) \| \| rs2235324 \| *TMPRSS6* \| G \| A \| NA \| Poggiali et al., 2015 \| Italy \| Associated with iron deficiecy \| (40) \| \| rs2280673 \| *TF* \| A \| C \| NA \| Benyamin, et al., 2009 \| Australian of European decent \| Elevated transferrin, decrease TSAT and ferritin \| (18) \| \| rs228904 \| *TMPRSS6* \| G \| A \| NA \| Tanaka et al., 2010 \| Italy and USA \| Significantly associated with elevated iron status \| (57) \| \| rs228916 \| *TMPRSS6* \| C \| T \| T \| Benyamin et al., 2014 \| European ancestry \| Reduced serum iron \| (5) \| \| rs228918 \| *TMPRSS6* \| A \| G \| C \| Chambers et al., 2009 \| European and Indian Ancestry \| Associated with decreased Hb levels, increased sTfR and low serum iron \| (21) \| \| rs228918 \| *TMPRSS6* \| A \| G \| G \| Gichohi-Wainaina et al., 2016 \| Kenyans, Tanzanians, S. Africans and African-Americans \| Reduced Hb \| (2) \| \| rs228921 \| *TMPRSS6* \| G \| A \| G \| Chambers et al., 2009 \| European and Indian Ancestry \| Associated with low iron status \| (21) \| \| rs228921 \| *TMPRSS6* \| G \| A \| G \| Gichohi-Wainaina et al., 2015 \| South Africa \| Higher sTfR and lower serum iron levels in combination with rs228918 \| (35) \|   **Table S3 Continued**   \| **SNPs** \| **Gene** \| **Minor allele** \| **Major allele** \| **Risk Allele** \| **Study/ first author** \| **Location/ Population/ Ethnicity^1^** \| **Associated trait^2^** \| **Reference^3^** \| \| --- \| --- \| --- \| --- \| --- \| --- \| --- \| --- \| --- \| \| rs2413450 \| *TMPRSS6* \| T \| C \| T \| Batar et al., 2018 \| Turkish \| Associated with elevated TIBC \| (47) \| \| rs2413450 \| *TMPRSS6* \| T \| C \| A \| Gichohi-Wainaina et al., 2016 \| Kenyans, Tanzanians, S. Africans and African-Americans \| Reduced Hb \| (2) \| \| rs2413450 \| *TMPRSS6* \| T \| C \| T \| Guo et al., 2016 \| Estonian \| Elevated MCH \| (63) \| \| rs2413450 \| *TMPRSS6* \| T \| C \| T \| Ganesh et al, 2009 \| European ancestry \| Reduced MCV, MCH and HCT \| (53) \| \| rs2543519 \| *TMPRSS6* \| G \| A \| NA \| Bhathia et al., 2017 \| India \| Associated with IRIDA \| (43) \| \| rs2543519 \| *TMPRSS6* \| G \| A \| NA \| Delbini et al., 2010 \| Italians \| Associated with iron deficiency \| (39) \| \| rs3811647 \| *TF* \| A \| G \| A \| Benyamin, et al., 2009 \| Australian of European dancestry \| Increased transferrin \| (18) \| \| rs3811647 \| *TF* \| A \| G \| A \| Pichler et al., 2011 \| Italy and USA \| Elevated transferrin concentration \| (6) \| \| rs3811647 \| *TF* \| A \| G \| A \| Blanco-Rojo et al., 2011 \| Spain \| Elevated serum transferritin \| (13) \| \| rs3811647 \| *TF* \| A \| G \| A \| ﻿Constantine et al., 2009 \| European ancestry \| Elevated serum transferritin \| (27) \| \| rs3811647 \| *TF* \| A \| G \| A \| An et al., 2012 \| Han Chinese \| Reduced Hb, increased transferrin and TIBC \| (32) \| \| rs3811647 \| *TF* \| A \| G \| A \| Benyamin et al., 2009 \| Aurtralians \| Raised serum iron and transferrin \| (18) \| \| rs3811647 \| *TF* \| A \| G \| A \| Gichohi-Wainaina et al., 2015 \| South Africa \| Heterozygotes are associated with lower sTfR and higher iron stores; homozygotes of both extremes have similar levels of sTfR and body iron stores \| (35) \| \| rs3811647 \| *TF* \| A \| G \| A \| Li et al., 2015 \| USA \| Elevated TIBC \| (12) \| \| rs3811647 \| *TF* \| A \| G \| A \| Koller et al., 2016 \| European-American (USA) \| Associated with TIBC serum iron \| (34) \| \| rs3811647 \| *TF* \| A \| G \| A \| McLaren et al., 2011 \| Americans of European ancentry \| Decreased TIBC and UIBC \| (33) \|   **Table S3 Continued**   \| **SNPs** \| **Gene** \| **Minor allele** \| **Major allele** \| **Risk Allele** \| **Study/ first author** \| **Location/ Population/ Ethnicity^1^** \| **Associated trait^2^** \| **Reference^3^** \| \| --- \| --- \| --- \| --- \| --- \| --- \| --- \| --- \| --- \| \| rs3811658 \| *TF* \| T \| C \| NA \| McLaren et al., 2012 \| Whites, African-Americans, Hispanics and Asians (US & Canada) \| Elevated TIBC in all the populations \| (30) \| \| rs3811658 \| *TF* \| T \| C \| T \| Gichohi-Wainaina et al., 2016 \| Kenyans, Tanzanians, S. Africans and African-Americans \| Increased Hb \| (2) \| \| rs3811658 \| *TF* \| T \| C \| NA \| ﻿Constantine et al., 2009 \| European ancestry \| Increased transferrin \| (27) \| \| rs4820268 \| *TMPRSS6* \| G \| A \| G \| Benyamin, et al., 2009 \| Australian of European ancestry \| Decreased serum iron and TSAT \| (18) \| \| rs4820268 \| *TMPRSS6* \| G \| A \| G \| Pichler et al., 2011 \| Italy and USA \| Decreased serum iron, Hb, MCV, MCH and ferritin; increased TF, sTfR and sTfR/ferritin index \| (6) \| \| rs4820268 \| *TMPRSS6* \| G \| A \| G \| Kullo et al., 2010 \| USA \| Associated with reduced MCH and MCHC \| (22) \| \| rs4820268 \| *TMPRSS6* \| G \| A \| G \| ﻿Constantine et al., 2009 \| European ancestry \| Lower TSAT and serum iron \| (27) \| \| rs4820268 \| *TMPRSS6* \| G \| A \| NA \| Chambers et al., 2009 \| European and Indian Ancestry \| Associated with decreased Hb levels, increased sTfR and low serum iron \| (21) \| \| rs4820268 \| *TMPRSS6* \| G \| A \| NA \| Delbini et al., 2010 \| Italians \| Associated with iron deficiency \| (39) \| \| rs4820268 \| *TMPRSS6* \| G \| A \| NA \| Tanaka et al., 2010 \| Italy and USA \| The C allele is significantly associated with increased serum iron and MCV, reduced RDW \| (57) \|   **Table S3 Continued**   \| **SNPs** \| **Gene** \| **Minor allele** \| **Major allele** \| **Risk Allele** \| **Study/ first author** \| **Location/ Population/ Ethnicity^1^** \| **Associated trait^2^** \| **Reference^3^** \| \| --- \| --- \| --- \| --- \| --- \| --- \| --- \| --- \| --- \| \| rs4820268 \| *TMPRSS6* \| G \| A \| G \| An et al., 2012 \| Han Chinese \| Low Hb, serum iron, TSAT. Associated with the risk of IDA \| (32) \| \| rs4820268 \| *TMPRSS6* \| G \| A \| NA \| Ji et al., 2018 \| Australia \| Associated with reduced ferritin levels \| (62) \| \| rs4820268 \| *TMPRSS6* \| G \| A \| NA \| Poggiali et al., 2015 \| Italy \| Associated with iron deficiecy \| (40) \| \| rs4820268 \| *TMPRSS6* \| G \| A \| NA \| Gan et al., 2012 \| Chinese \| Associated with low Hb and ferritin \| (46) \| \| rs4820268 \| *TMPRSS6* \| G \| A \| A \| Li et al., 2015 \| USA \| Elevated serum iron and TSAT \| (12) \| \| rs4820268 \| *TMPRSS6* \| G \| A \| G \| Piao et al., 2017 \| Chinese adolescents \| G alleles associated with lower serum ferritin \| (31) \| \| rs4820268 \| *TMPRSS6* \| G \| A \| G \| Gichohi-Wainaina et al., 2016 \| Kenyans, Tanzanians, S. Africans and African-Americans \| Reduced Hb \| (2) \| \| rs5756504 \| *T­­­MPRSS6* \| T \| C \| T \| Kullo et al., 2010 \| USA \| Elevated MCH \| (22) \| \| rs5756504 \| *TMPRSS6* \| T \| C \| NA \| Tanaka et al., 2010 \| Italy and USA \| The T allele Asscoiated with significant increase in serum iron levels \| (57) \| \| rs5756504 \| *TMPRSS6* \| T \| C \| T \| Seiki et al., 2018 \| Japanese \| Associated with elevated MCV, MCH and MCHC \| (60) \| \| rs5756504 \| *TMPRSS6* \| T \| C \| NA \| Kamatani et al., 2010 \| Japanese \| The T allele are asscoiated with elevated Hb \| (56) \| \| rs5756506 \| *TMPRSS6* \| C \| G \| NA \| ﻿Constantine et al., 2009 \| European ancestry \| increase serum iron and TSAT \| (27) \| \| rs5756506 \| *TMPRSS6* \| C \| G \| C \| Seiki et al., 2018 \| Japanese \| Elevated MCH, Hb \| (60) \| \| rs5756506 \| *TMPRSS6* \| C \| G \| NA \| Batar et al., 2018 \| Turkey \| Associated with elevated Hb and HCT \| (47) \| \| rs5756512 \| *TMPRSS6* \| T \| C \| NA \| Bhathia et al., 2017 \| India \| Associated with IRIDA \| (43) \| \| rs5756516 \| *TMPRSS6* \| T \| C \| NA \| Bhathia et al., 2017 \| India \| Associated with IRIDA \| (43) \|   **Table S3 Continued**   \| **SNPs** \| **Gene** \| **Minor allele** \| **Major allele** \| **Risk Allele** \| **Study/ first author** \| **Location/ Population/ Ethnicity^1^** \| **Associated trait^2^** \| **Reference^3^** \| \| --- \| --- \| --- \| --- \| --- \| --- \| --- \| --- \| --- \| \| rs732756 \| *TMPRSS6* \| C \| T \| NA \| Bhathia et al., 2017 \| India \| Associated with IRIDA \| (43) \| \| rs7385804 \| *TFR2* \| C \| A \| C \| Pichler et al., 2011 \| Italy and USA \| Increased serum iron \| (6) \| \| rs7385804 \| *TFR2* \| C \| A \| C \| Benyamin et al., 2014 \| European ancestry \| Elevated iron, TSAT and ferritin, and reduced tranferrin \| (5) \| \| rs7385804 \| *TFR2* \| C \| A \| C \| Soranzo et al., 2009 \| Europeans and South Asians \| Elevated RBC \| (37) \| \| rs7385804 \| *TFR2* \| C \| A \| C \| An et al., 2012 \| Han Chinese \| Lower TSAT and serum iron \| (32) \| \| rs7385804 \| *TFR2* \| C \| A \| C \| Gichohi-Wainaina et al., 2016 \| Kenyans, Tanzanians, S. Africans and African-Americans \| Increased ferritin in Kenyans \| (2) \| \| rs744653 \| *SLC40A1* \| C \| T \| T \| Benyamin et al., 2014 \| European ancestry \| Moderated HH via elevated transferrin and reduced TSAT, and ferritin levels \| (5) \| \| rs7638018 \| *TF* \| G \| A \| NA \| McLaren et al., 2012 \| Whites, African-Americans, Hispanics and Asians (US & Canada) \| Increased TIBC in all the populations \| (30) \| \| rs78174698 \| *TMPRSS6* \| A \| G \| NA \| Bhathia et al., 2017 \| India \| Associated with IRIDA \| (43) \| \| rs8177179 \| *TF* \| G \| A \| A \| Benyamin et al., 2014 \| European ancestry \| Reduced transferrin \| (5) \| \| rs8177248 \| *TF* \| T \| C \| NA \| McLaren et al., 2012 \| Whites, African-Americans, Hispanics and Asians (US & Canada) \| increased TIBC in all the populations \| (30) \| \| rs732756 \| *TMPRSS6* \| C \| T \| NA \| Bhathia et al., 2017 \| India \| Associated with IRIDA \| (12) \|   **Table S3 Continued**   \| **SNPs** \| **Gene** \| **Minor allele** \| **Major allele** \| **Risk Allele** \| **Study/ first author** \| **Location/ Population/ Ethnicity^1^** \| **Associated trait^2^** \| **Reference^3^** \| \| --- \| --- \| --- \| --- \| --- \| --- \| --- \| --- \| --- \| \| rs855788 \| *TMPRSS6* \| A \| G \| NA \| Tanaka et al., 2010 \| Italy and USA \| Significantly associated with elevated iron status \| (57) \| \| rs855791 \| *TMPRSS6* \| A \| G \| A \| Lee et al., 2012 \| White Americans \| No significant assciation with outcome variables \| (38) \| \| rs855791 \| *TMPRSS6* \| A \| G \| T \| Pei et al., 2014 \| Taiwan \| Associated with IDA; C alleles protective against IDA \| (50) \| \| rs855791 \| *TMPRSS6* \| A \| G \| A \| Athiyarath et al., 2015 \| India \| Significantly associated with higher levels of serum iron \| (10) \| \| rs855791 \| *TMPRSS6* \| A \| G \| A \| Kullo et al., 2010 \| USA \| Significantly associated with decreased MCV, MCH and MCHC \| (22) \| \| rs855791 \| *TMPRSS6* \| A \| G \| A \| Valenti et al., 2012 \| Italians \| Lower MCV, ferritin and elevated hepcidin \| (58) \| \| rs855791 \| *TMPRSS6* \| A \| G \| A \| Beutler et al., 2010 \| European ancestry \| Associated with iron deficiency \| (51) \| \| rs855791 \| *TMPRSS6* \| A \| G \| A \| Pelusi et al., 2013 \| Italy \| Associated with elevated hepcidin; low MCV \| (52) \| \| rs855791 \| *TMPRSS6* \| A \| G \| A \| Chambers et al., 2009 \| European and Indian Ancestry \| Associated with decreased Hb levels, increased sTfR and low serum iron \| (21) \| \| rs855791 \| *TMPRSS6* \| A \| G \| NA \| Bhathia et al., 2017 \| India \| Associated with IRIDA \| (43) \| \| rs855791 \| *TMPRSS6* \| A \| G \| NA \| Delbini et al., 2010 \| Italy \| Associated with iron deficiency \| (39) \| \| rs855791 \| *TMPRSS6* \| A \| G \| NA \| Tanaka et al., 2010 \| Italy and USA \| The A allele is significantly associated with increased serum iron and Hb, MCV, reduced RDW \| (57) \| \| rs855791 \| *TMPRSS6* \| A \| G \| NA \| Galesloot et al., 2013 \| Netherlands \| Significantly associated with serum iron and TSAT \| (15) \|   **Table S3 continued**   \| **SNPs** \| **Gene** \| **Minor allele** \| **Major allele** \| **Risk Allele** \| **Study/ first author** \| **Location/ Population/ Ethnicity^1^** \| **Associated trait^2^** \| **Reference^3^** \| \| --- \| --- \| --- \| --- \| --- \| --- \| --- \| --- \| --- \| \| rs855791 \| *TMPRSS6* \| A \| G \| A \| An et al., 2012 \| Han Chinese \| Low Hb, serum iron, TSAT. Associated with the risk of IDA \| (32) \| \| rs855791 \| *TMPRSS6* \| A \| G \| A \| Benyamin et al., 2009 \| Aurtralians \| Reduced serum iron, TSAT, ferritin, Hb and MCV, and raised transferrin \| (18) \| \| rs855791 \| *TMPRSS6* \| A \| G \| A \| van der Harst et al., 2012 \| Europeans and South Asians \| Elevated MCH \| (54) \| \| rs855791 \| *TMPRSS6* \| A \| G \| NA \| Kamatani et al., 2010 \| Japanese \| The G alleles are associated with elevated MCV, MCH and MCHC \| (56) \| \| rs855791 \| *TMPRSS6* \| A \| G \| NA \| Batar et al., 2018 \| Turkey \| Associated with increased RBC count \| (47) \| \| rs855791 \| *TMPRSS6* \| A \| G \| NA \| Poggiali et al., 2015 \| Italy \| Associated with low Hb, MCV and MCH \| (40) \| \| rs855791 \| *TMPRSS6* \| A \| G \| NA \| Gan et al., 2012 \| Chinese \| Associated with low Hb and ferritin \| (46) \| \| rs855791 \| *TMPRSS6* \| A \| G \| A \| Nai et al., 2011 \| USA \| Associated with elevated hepcidin, hepcidin/TSAT ratio, hepcidin/ferritin ratio, and low TSAT and serum iron \| (48) \| \| rs855791 \| *TMPRSS6* \| A \| G \| A \| Cheng et al., 2014 \| Australia \| G alleles associated with higher serum iron and lower hepcidin at baseline \| (59) \| \| rs855791 \| *TMPRSS6* \| A \| G \| NA \| De Falco et al., 2018 \| Italy \| Significanty associated with IDA \| (16) \| \| rs855791 \| *TMPRSS6* \| A \| G \| T \| Sorensen et al., 2015 \| Denmark \| The T allele is associated with lower iron stores in men \| (8) \| \| rs855791 \| *TMPRSS6* \| A \| G \| A \| Benyamin et al., 2014 \| European ancestry \| Reduced serum iron, TSAT and ferritin, and elevated tranferrin \| (5) \|   **Table S3 continued**   \| **SNPs** \| **Gene** \| **Minor allele** \| **Major allele** \| **Risk Allele** \| **Study/ first author** \| **Location/ Population/ Ethnicity^1^** \| **Associated trait^2^** \| **Reference^3^** \| \| --- \| --- \| --- \| --- \| --- \| --- \| --- \| --- \| --- \| \| rs855791 \| *TMPRSS6* \| A \| G \| A \| Pichler et al., 2013 \| European Ancestry populations \| Protective against iron overload \| (17) \| \| rs855791 \| *TMPRSS6* \| A \| G \| A \| Traglia et al., 2011 \| Italians \| Non-statistical significant increase in hepcidin/ferritin ratio and non-significant decrease in hepcidin and ferritin \| (45) \| \| rs855791 \| *TMPRSS6* \| A \| G \| A \| Danquah et al., 2014 \| Rwanda \| Non-significantly associated with low Hb \| (49) \| \| rs855791 \| *TMPRSS6* \| A \| G \| A \| Bedard et al., 2018 \| UK \| Associated with reduced iron stores \| (55) \| \| rs9610643 \| *TMPRSS6* \| A \| G \| NA \| Bhathia et al., 2017 \| India \| Associated with IRIDA \| (43) \| \| rs9872999 \| *TF* \| C \| T \| NA \| Li et al., 2015 \| USA \| Reduced TIBC \| (12) \| |
| --- | --- | --- | --- | --- | --- | --- | --- | --- | --- | --- | --- | --- | --- | --- | --- | --- | --- | --- | --- | --- | --- | --- | --- | --- | --- | --- | --- | --- | --- | --- | --- | --- | --- | --- | --- | --- | --- | --- | --- | --- | --- | --- | --- | --- | --- | --- | --- | --- | --- | --- | --- | --- | --- | --- | --- | --- | --- | --- | --- | --- | --- | --- | --- | --- | --- | --- | --- | --- | --- | --- | --- | --- | --- | --- | --- | --- | --- | --- | --- | --- | --- | --- | --- | --- | --- | --- | --- | --- | --- | --- | --- | --- | --- | --- | --- | --- | --- | --- | --- | --- | --- | --- | --- | --- | --- | --- | --- | --- | --- | --- | --- | --- | --- | --- | --- | --- | --- | --- | --- | --- | --- | --- | --- | --- | --- | --- | --- | --- | --- | --- | --- | --- | --- | --- | --- | --- | --- | --- | --- | --- | --- | --- | --- | --- | --- | --- | --- | --- | --- | --- | --- | --- | --- | --- | --- | --- | --- | --- | --- | --- | --- | --- | --- | --- | --- | --- | --- | --- | --- | --- | --- | --- | --- | --- | --- | --- | --- | --- | --- | --- | --- | --- | --- | --- | --- | --- | --- | --- | --- | --- | --- | --- | --- | --- | --- | --- | --- | --- | --- | --- | --- | --- | --- | --- | --- | --- | --- | --- | --- | --- | --- | --- | --- | --- | --- | --- | --- | --- | --- | --- | --- | --- | --- | --- | --- | --- | --- | --- | --- | --- | --- | --- | --- | --- | --- | --- | --- | --- | --- | --- | --- | --- | --- | --- | --- | --- | --- | --- | --- | --- | --- | --- | --- | --- | --- | --- | --- | --- | --- | --- | --- | --- | --- | --- | --- | --- | --- | --- | --- | --- | --- | --- | --- | --- | --- | --- | --- | --- | --- | --- | --- | --- | --- | --- | --- | --- | --- | --- | --- | --- | --- | --- | --- | --- | --- | --- | --- | --- | --- | --- | --- | --- | --- | --- | --- | --- | --- | --- | --- | --- | --- | --- | --- | --- | --- | --- | --- | --- | --- | --- | --- | --- | --- | --- | --- | --- | --- | --- | --- | --- | --- | --- | --- | --- | --- | --- | --- | --- | --- | --- | --- | --- | --- | --- | --- | --- | --- | --- | --- | --- | --- | --- | --- | --- | --- | --- | --- | --- | --- | --- | --- | --- | --- | --- | --- | --- | --- | --- | --- | --- | --- | --- | --- | --- | --- | --- | --- | --- | --- | --- | --- | --- | --- | --- | --- | --- | --- | --- | --- | --- | --- | --- | --- | --- | --- | --- | --- | --- | --- | --- | --- | --- | --- | --- | --- | --- | --- | --- | --- | --- | --- | --- | --- | --- | --- | --- | --- | --- | --- | --- | --- | --- | --- | --- | --- | --- | --- | --- | --- | --- | --- | --- | --- | --- | --- | --- | --- | --- | --- | --- | --- | --- | --- | --- | --- | --- | --- | --- | --- | --- | --- | --- | --- | --- | --- | --- | --- | --- | --- | --- | --- | --- | --- | --- | --- | --- | --- | --- | --- | --- | --- | --- | --- | --- | --- | --- | --- | --- | --- | --- | --- | --- | --- | --- | --- | --- | --- | --- | --- | --- | --- | --- | --- | --- | --- | --- | --- | --- | --- | --- | --- | --- | --- | --- | --- | --- | --- | --- | --- | --- | --- | --- | --- | --- | --- | --- | --- | --- | --- | --- | --- | --- | --- | --- | --- | --- | --- | --- | --- | --- | --- | --- | --- | --- | --- | --- | --- | --- | --- | --- | --- | --- | --- | --- | --- | --- | --- | --- | --- | --- | --- | --- | --- | --- | --- | --- | --- | --- | --- | --- | --- | --- | --- | --- | --- | --- | --- | --- | --- | --- | --- | --- | --- | --- | --- | --- | --- | --- | --- | --- | --- | --- | --- | --- | --- | --- | --- | --- | --- | --- | --- | --- | --- | --- | --- | --- | --- | --- | --- | --- | --- | --- | --- | --- | --- | --- | --- | --- | --- | --- | --- | --- | --- | --- | --- | --- | --- | --- | --- | --- | --- | --- | --- | --- | --- | --- | --- | --- | --- | --- | --- | --- | --- | --- | --- | --- | --- | --- | --- | --- | --- | --- | --- | --- | --- | --- | --- | --- | --- | --- | --- | --- | --- | --- | --- | --- | --- | --- | --- | --- | --- | --- | --- | --- | --- | --- | --- | --- | --- | --- | --- | --- | --- | --- | --- | --- | --- | --- | --- | --- | --- | --- | --- | --- | --- | --- | --- | --- | --- | --- | --- | --- | --- | --- | --- | --- | --- | --- | --- | --- | --- | --- | --- | --- | --- | --- | --- | --- | --- | --- | --- | --- | --- | --- | --- | --- | --- | --- | --- | --- | --- | --- | --- | --- | --- | --- | --- | --- | --- | --- | --- | --- | --- | --- | --- | --- | --- | --- | --- | --- | --- | --- | --- | --- | --- | --- | --- | --- | --- | --- | --- | --- | --- | --- | --- | --- | --- | --- | --- | --- | --- | --- | --- | --- | --- | --- | --- | --- | --- | --- | --- | --- | --- | --- | --- | --- | --- | --- | --- | --- | --- | --- | --- | --- | --- | --- | --- | --- | --- | --- | --- | --- | --- | --- | --- | --- | --- | --- | --- | --- | --- | --- | --- | --- | --- | --- | --- | --- | --- | --- | --- | --- | --- | --- | --- | --- | --- | --- | --- | --- | --- | --- | --- | --- | --- | --- | --- | --- | --- | --- | --- | --- | --- | --- | --- | --- | --- | --- | --- | --- | --- | --- | --- | --- | --- | --- | --- | --- | --- | --- | --- | --- | --- | --- | --- | --- | --- | --- | --- | --- | --- | --- | --- | --- | --- | --- | --- | --- | --- | --- | --- | --- | --- | --- | --- | --- | --- | --- | --- | --- | --- | --- | --- | --- | --- | --- | --- | --- | --- | --- | --- | --- | --- | --- | --- | --- | --- | --- | --- | --- | --- | --- | --- | --- | --- | --- | --- | --- | --- | --- | --- | --- | --- | --- | --- | --- | --- | --- | --- | --- | --- | --- | --- | --- | --- | --- | --- | --- | --- | --- | --- | --- | --- | --- | --- | --- | --- | --- | --- | --- | --- | --- | --- | --- | --- | --- | --- | --- | --- | --- | --- | --- | --- | --- | --- | --- | --- | --- | --- | --- | --- | --- | --- | --- | --- | --- | --- | --- | --- | --- | --- | --- | --- | --- | --- | --- | --- | --- | --- | --- | --- | --- | --- | --- | --- | --- | --- | --- | --- | --- | --- | --- | --- | --- | --- | --- | --- | --- | --- | --- | --- | --- | --- | --- | --- | --- | --- | --- | --- | --- | --- | --- | --- | --- | --- | --- | --- | --- | --- | --- | --- | --- | --- | --- | --- | --- | --- | --- | --- | --- | --- | --- | --- | --- | --- | --- | --- | --- | --- | --- | --- | --- | --- | --- | --- | --- | --- | --- | --- | --- | --- | --- | --- | --- | --- | --- | --- | --- | --- | --- | --- | --- | --- | --- | --- | --- | --- | --- | --- | --- | --- | --- | --- | --- | --- | --- | --- | --- | --- | --- | --- | --- | --- | --- | --- | --- | --- | --- | --- | --- | --- | --- | --- | --- | --- | --- | --- | --- | --- | --- | --- | --- | --- | --- | --- | --- | --- | --- | --- | --- | --- | --- | --- | --- | --- | --- | --- | --- | --- | --- | --- | --- | --- | --- | --- | --- | --- | --- | --- | --- | --- | --- | --- | --- | --- | --- | --- | --- | --- | --- | --- | --- | --- | --- | --- | --- | --- | --- | --- | --- | --- | --- | --- | --- | --- | --- | --- | --- | --- | --- | --- | --- | --- | --- | --- | --- | --- | --- | --- | --- | --- | --- | --- | --- | --- | --- | --- | --- | --- | --- | --- | --- | --- | --- | --- | --- | --- | --- | --- | --- | --- | --- | --- | --- | --- | --- | --- | --- | --- | --- | --- | --- | --- | --- | --- | --- | --- | --- | --- | --- | --- | --- | --- | --- | --- | --- | --- | --- | --- | --- | --- | --- | --- | --- | --- | --- | --- | --- | --- | --- | --- | --- | --- | --- | --- | --- | --- | --- | --- | --- | --- | --- | --- | --- | --- | --- | --- | --- | --- | --- | --- | --- | --- | --- | --- | --- | --- | --- | --- | --- | --- | --- | --- | --- | --- | --- | --- | --- | --- | --- | --- | --- | --- | --- | --- | --- | --- | --- | --- | --- | --- | --- | --- | --- | --- | --- | --- | --- | --- | --- | --- | --- | --- | --- | --- | --- | --- | --- | --- | --- | --- | --- | --- | --- | --- | --- | --- | --- | --- | --- | --- | --- | --- | --- | --- | --- | --- | --- | --- | --- | --- | --- | --- | --- | --- | --- | --- | --- | --- | --- | --- | --- | --- | --- | --- | --- | --- | --- | --- | --- | --- | --- | --- | --- | --- | --- | --- | --- | --- | --- | --- | --- | --- | --- | --- | --- | --- | --- | --- | --- | --- | --- | --- | --- | --- | --- | --- | --- | --- | --- | --- | --- | --- | --- | --- | --- | --- | --- | --- | --- | --- | --- | --- | --- | --- | --- | --- | --- | --- | --- | --- | --- | --- | --- | --- | --- | --- | --- | --- | --- | --- | --- | --- | --- | --- | --- | --- | --- | --- | --- | --- | --- | --- | --- | --- | --- | --- | --- | --- | --- | --- | --- | --- | --- | --- | --- | --- | --- | --- | --- | --- | --- | --- | --- | --- | --- | --- | --- | --- | --- | --- | --- | --- | --- | --- | --- | --- | --- | --- | --- | --- | --- | --- | --- | --- | --- | --- | --- | --- | --- | --- | --- | --- | --- | --- | --- | --- | --- | --- | --- | --- | --- | --- | --- | --- | --- | --- | --- | --- | --- | --- | --- | --- | --- | --- | --- | --- | --- | --- | --- | --- | --- | --- | --- | --- | --- | --- | --- | --- | --- | --- | --- | --- | --- | --- | --- | --- | --- | --- | --- | --- | --- | --- | --- | --- | --- | --- | --- | --- | --- | --- | --- | --- | --- | --- | --- | --- | --- | --- | --- | --- | --- | --- | --- | --- | --- | --- | --- | --- | --- | --- | --- | --- | --- | --- | --- | --- | --- | --- | --- | --- | --- | --- | --- | --- | --- | --- | --- | --- | --- | --- | --- | --- | --- | --- | --- | --- | --- | --- | --- | --- | --- | --- | --- | --- | --- | --- | --- | --- | --- | --- | --- | --- | --- | --- | --- | --- | --- | --- | --- | --- | --- | --- | --- | --- | --- | --- | --- | --- | --- | --- | --- | --- | --- | --- | --- | --- | --- | --- | --- | --- | --- | --- | --- | --- | --- | --- | --- | --- | --- | --- | --- | --- | --- | --- | --- | --- | --- | --- | --- | --- | --- | --- | --- | --- | --- | --- | --- | --- | --- | --- | --- | --- | --- | --- | --- | --- | --- | --- | --- | --- | --- | --- | --- | --- | --- | --- | --- | --- | --- | --- | --- | --- | --- | --- | --- | --- | --- | --- | --- | --- | --- | --- | --- | --- | --- | --- | --- | --- | --- | --- | --- | --- |

Hb, haemoglobin; HCT, haematocrit; HFE, High fe; HH, hereditary hemochromatosis; IDA, iron deficiency anaemia; MCH, mean corpuscular haemoglobin; NA, not available; SAS, South Asians; SI, serum iron; *SLC40A1*, solute carrier family 40 member 1; SNP, single nucleotide polymorphism; sTfR, soluble transferrin receptor

NA indicates SNPs that we could not establish the risk allele because it was not stated by the respective studies that reported the SNPs.

^1^The population or the study location where the study that reported each SNP was conducted.

^2^The phenotype that was reported

^3^The study that reported each SNP

**Reference:**

1. Andreani M, Radio FC, Testi M, De Bernardo C, Troiano M, Majore S, et al. Association of hepcidin promoter c.-582 A>G variant and iron overload in thalassemia major. Haematologica. 2009;94(9):1293–6.

2. Gichohi-Wainaina WN, Tanaka T, Towers GW, Verhoef H, Veenemans J, Talsma EF, et al. Associations between Common Variants in Iron-Related Genes with Haematological Traits in Populations of African Ancestry. PLoS One [Internet]. 2016;11(6):e0157996. Available from: http://www.ncbi.nlm.nih.gov/pubmed/27332551

3. Javaheri-Kermani M, Farazmandfar T, Ajami A, Yazdani Y. Impact of hepcidin antimicrobial peptide on iron overload in tuberculosis patients. Scand J Infect Dis [Internet]. 2014;46(10):693–6. Available from: http://informahealthcare.com/doi/abs/10.3109/00365548.2014.929736

4. Radio FC, Majore S, Aurizi C, Sorge F, Biolcati G, Bernabini S, et al. Hereditary hemochromatosis type 1 phenotype modifiers in Italian patients. The controversial role of variants in HAMP, BMP2, FTL and SLC40A1 genes. Blood Cells Mol Dis [Internet]. 2015 Jun;55(1):71–5. Available from: http://dx.doi.org/10.1016/j.bcmd.2015.04.001

5. Benyamin B, Esko T, Ried JS, Radhakrishnan A, Vermeulen SH, Traglia M, et al. Novel loci affecting iron homeostasis and their effects in individuals at risk for hemochromatosis. Nat Commun [Internet]. 2014 Oct 29;5(2):4926. Available from: http://www.ncbi.nlm.nih.gov/pubmed/25352340

6. Pichler I, Minelli C, Sanna S, Tanaka T, Schwienbacher C, Naitza S, et al. Identification of a common variant in the TFR2 gene implicated in the physiological regulation of serum iron levels. Hum Mol Genet [Internet]. 2011 Mar 15;20(6):1232–40. Available from: http://www.ncbi.nlm.nih.gov/pubmed/21208937

7. Garewal G, Das R, Ahluwalia J, Marwaha RK. Prevalence of the H63D mutation of the HFE in north India: Its presence does not cause iron overload in beta thalassemia trait. Eur J Haematol. 2005;74(4):333–6.

8. Sørensen E, Rigas AS, Thørner LW, Burgdorf KS, Pedersen OB, Petersen MS, et al. Genetic factors influencing ferritin levels in 14,126 blood donors: Results from the Danish Blood Donor Study. Transfusion. 2016;56(3):622–7.

9. Mast AE, Lee T-H, Schlumpf KS, Wright DJ, Johnson B, Carrick DM, et al. The impact of HFE mutations on haemoglobin and iron status in individuals experiencing repeated iron loss through blood donation*. Br J Haematol [Internet]. 2012 Feb;156(3):388–401. Available from: http://www.ncbi.nlm.nih.gov/pubmed/22118647

10. Athiyarath R, Shaktivel K, Abraham V, Singh D, Bondu JD, Chapla A, et al. Association of genetic variants with response to iron supplements in pregnancy. Genes Nutr [Internet]. 2015 Jul 30;10(4):25. Available from: http://link.springer.com/10.1007/s12263-015-0474-2

11. Whitfield JB, Cullen LM, Jazwinska EC, Powell LW, Heath AC, Zhu G, et al. Effects of HFE C282Y and H63D polymorphisms and polygenic background on iron stores in a large community sample of twins. Am J Hum Genet [Internet]. 2000 Apr;66(4):1246–58. Available from: http://www.ncbi.nlm.nih.gov/pubmed/10739755

12. Li J, Lange LA, Duan Q, Lu Y, Singleton AB, Zonderman AB, et al. Genome-wide admixture and association study of serum iron, ferritin, transferrin saturation and total iron binding capacity in African Americans. Hum Mol Genet. 2015;24(2):572–81.

13. Blanco-Rojo R, Baeza-Richer C, López-Parra AM, Pérez-Granados AM, Brichs A, Bertoncini S, et al. Four variants in transferrin and HFE genes as potential markers of iron deficiency anaemia risk: an association study in menstruating women. Nutr Metab (Lond) [Internet]. 2011 Oct 6;8:69. Available from: http://www.ncbi.nlm.nih.gov/pubmed/21978626

14. Beutler E, Felitti V, Gelbart T, Waalen J. Haematological effects of the C282Y HFE mutation in homozygous and heterozygous states among subjects of northern and southern European ancestry. Br J Haematol. 2003;120(5):887–93.

15. Galesloot TE, Geurts-Moespot AJ, den Heijer M, Sweep FCGJ, Fleming RE, Kiemeney L a LM, et al. Associations of common variants in HFE and TMPRSS6 with iron parameters are independent of serum hepcidin in a general population: a replication study. J Med Genet [Internet]. 2013;50(9):593–8. Available from: http://www.ncbi.nlm.nih.gov/pubmed/23794717

16. De Falco L, Tortora R, Imperatore N, Bruno M, Capasso M, Girelli D, et al. The role of TMPRSS6 and HFE variants in iron deficiency anemia in celiac disease. Am J Hematol. 2018;93(3):383–93.

17. Pichler I, Del Greco M F, Gögele M, Lill CM, Bertram L, Do CB, et al. Serum iron levels and the risk of Parkinson disease: a Mendelian randomization study. PLoS Med [Internet]. 2013;10(6):e1001462. Available from: http://www.ncbi.nlm.nih.gov/pubmed/23750121

18. Benyamin B, McRae AF, Zhu G, Gordon S, Henders AK, Palotie A, et al. Variants in TF and HFE explain approximately 40% of genetic variation in serum-transferrin levels. Am J Hum Genet [Internet]. 2009 Jan;84(1):60–5. Available from: http://dx.doi.org/10.1016/j.ajhg.2008.11.011

19. Gordeuk VR, Brannon PM. Ethnic and genetic factors of iron status in women of reproductive age. Am J Clin Nutr. 2017;106:1594S-1599S.

20. Blanco-Rojo R, Toxqui L, López-Parra AM, Baeza-Richer C, Pérez-Granados AM, Arroyo-Pardo E, et al. Influence of diet, menstruation and genetic factors on iron status: A cross-sectional study in Spanish women of childbearing age. Int J Mol Sci. 2014;15(3):4077–87.

21. Chambers JC, Zhang W, Li Y, Sehmi J, Wass MN, Zabaneh D, et al. Genome-wide association study identifies variants in TMPRSS6 associated with hemoglobin levels. Nat Genet [Internet]. 2009 Nov;41(11):1170–2. Available from: http://www.ncbi.nlm.nih.gov/pubmed/19820698

22. Kullo IJ, Ding K, Jouni H, Smith CY, Chute CG. A Genome-Wide Association Study of Red Blood Cell Traits Using the Electronic Medical Record. 2010;5(9):1–9.

23. Chen Z, Tang H, Qayyum R, Schick UM, Nalls MA, Handsaker R, et al. Genome-wide association analysis of red blood cell traits in African Americans: The cogent network. Hum Mol Genet. 2013;22(12):2529–38.

24. Kasvosve I, Gomo ZAR, Nathoo KJ, Matibe P, Mudenge B, Loyevsky M, et al. Effect of ferroportin Q248H polymorphism on iron status in African children. 2018;(April):1102–6.

25. Masaisa F, Breman C, Gahutu JB, Mukiibi J, Delanghe J, Philippé J. Ferroportin (SLC40A1) Q248H mutation is associated with lower circulating serum hepcidin levels in Rwandese HIV-positive women. Ann Hematol. 2012;91(6):911–6.

26. Rivers CA, Barton JC, Gordeuk VR, Acton RT, Speechley MR, Snively BM, et al. Association of ferroportin Q248H polymorphism with elevated levels of serum ferritin in African Americans in the Hemochromatosis and Iron Overload Screening (HEIRS) Study. Blood Cells, Mol Dis. 2007;38(3):247–52.

27. Constantine CC, Anderson GJ, Vulpe CD, Mclaren CE, Bahlo M, Yeap HL, et al. A novel association between a SNP in CYBRD1 and serum ferritin levels in a cohort study of HFE hereditary haemochromatosis. 2009;(August):140–9.

28. Sarria B, Lopez-parra AM, Perez-granados AM, Arroyo-pardo E, Roe MA, Teucher B, et al. The G277S transferrin mutation does not affect iron absorption in iron deficient women. 2007;57–60.

29. Lee PL, Halloran C, Trevino R, Felitti V, Beutler E. Human transferrin G277S mutation: a risk factor for iron deficiency anaemia. Br J Haematol [Internet]. 2001 Nov;115(2):329–33. Available from: http://www.ncbi.nlm.nih.gov/pubmed/11703331

30. McLaren CE, McLachlan S, Garner CP, Vulpe CD, Gordeuk VR, Eckfeldt JH, et al. Associations between single nucleotide polymorphisms in iron-related genes and iron status in multiethnic populations. PLoS One. 2012;7(6).

31. Piao W, Wang L, Zhang T, Wang Z, Shangguan S, Sun J, et al. A single-nucleotide polymorphism in transferrin is associated with soluble transferrin receptor in Chinese adolescents. Asia Pac J Clin Nutr. 2017;26(6):1170–8.

32. An P, Wu Q, Wang H, Guan Y, Mu M, Liao Y, et al. TMPRSS6, but not TF, TFR2 or BMP2 variants are associated with increased risk of iron-deficiency anemia. Hum Mol Genet. 2012;21(9):2124–31.

33. McLaren CE, Garner CP, Constantine CC, McLachlan S, Vulpe CD, Snively BM, et al. Genome-wide association study identifies genetic loci associated with iron deficiency. PLoS One. 2011;6(3).

34. Koller DL, Imel EA, Lai D, Padgett LR, Acton D, Gray A, et al. Genome-wide association study of serum iron phenotypes in premenopausal women of European descent. Blood Cells, Mol Dis [Internet]. 2016;57:50–3. Available from: http://dx.doi.org/10.1016/j.bcmd.2015.12.002

35. Gichohi-Wainaina, W. N. Melse-Boonstra, A. Swinkels, D. W. Zimmermann, M. B. Feskens, E. J. Towers GW. Common variants and haplotypes in the TF, TNF- alpha , and TMPRSS6 genes are associated with iron status in a female black South. J Nutr 2015. 2015;145(5):945–53.

36. Benyamin B, Ferreira MAR, Willemsen G, Gordon S, Middelberg RPS, McEvoy BP, et al. Common variants in TMPRSS6 are associated with iron status and erythrocyte volume. Nat Genet [Internet]. 2009 Nov 11;41(11):1173–5. Available from: http://www.nature.com/doifinder/10.1038/ng.456

37. Soranzo N, Spector TD, Mangino M, Kühnel B, Rendon A, Teumer A, et al. A genome-wide meta-analysis identifies 22 loci associated with eight hematological parameters in the HaemGen consortium. Nat Genet [Internet]. 2009;41(11):1182–90. Available from: http://dx.doi.org/10.1038/ng.467

38. Lee PL, Barton JC, Khaw PL, Bhattacharjee SY, Barton JC. Common TMPRSS6 mutations and iron, erythrocyte, and pica phenotypes in 48 women with iron deficiency or depletion. Blood Cells, Mol Dis [Internet]. 2012 Feb;48(2):124–7. Available from: http://dx.doi.org/10.1016/j.bcmd.2011.12.003

39. Delbini P, Vaja V, Graziadei G, Duca L, Nava I, Refaldi C, et al. Genetic variability of TMPRSS6 and its association with iron deficiency anaemia. Br J Haematol. 2010;151(3):281–4.

40. Poggiali E, Andreozzi F, Nava I, Consonni D, Graziadei G, Cappellini MD. The role of TMPRSS6 polymorphisms in iron deficiency anemia partially responsive to oral iron treatment. Am J Hematol. 2015;90(4):306–9.

41. Galesloot TE, Verweij N, Traglia M, Barbieri C, Van Dijk F, Geurts-Moespot AJ, et al. Meta-GWAS and meta-analysis of exome array studies do not reveal genetic determinants of serum hepcidin. PLoS One. 2016;11(11):1–13.

42. Kloss-Brandstätter A, Erhart G, Lamina C, Meister B, Haun M, Coassin S, et al. Candidate gene sequencing of SLC11A2 and TMPRSS6 in a family with severe anaemia: Common SNPs, rare haplotypes, no causative mutation. PLoS One. 2012;7(4):1–8.

43. Bhatia P, Singh A, Hegde A, Jain R, Bansal D. Systematic evaluation of paediatric cohort with iron refractory iron deficiency anaemia (IRIDA) phenotype reveals multiple TMPRSS6 gene variations. Br J Haematol [Internet]. 2017 Apr;177(2):311–8. Available from: http://doi.wiley.com/10.1111/bjh.14554

44. Valenti L, Fracanzani AL, Rametta R, Fraquelli M, Soverini G, Pelusi S, et al. Effect of the A736V TMPRSS6 polymorphism on the penetrance and clinical expression of hereditary hemochromatosis. J Hepatol [Internet]. 2012;57(6):1319–25. Available from: http://dx.doi.org/10.1016/j.jhep.2012.07.041

45. Traglia M, Girelli D, Biino G, Campostrini N, Corbella M, Sala C, et al. Association of HFE and TMPRSS6 genetic variants with iron and erythrocyte parameters is only in part dependent on serum hepcidin concentrations. J Med Genet [Internet]. 2011 Sep;48(9):629–34. Available from: http://www.ncbi.nlm.nih.gov/pubmed/21785125

46. Gan W, Guan Y, Wu Q, An P, Zhu J, Lu L, et al. Association of TMPRSS6 polymorphisms with ferritin, hemoglobin, and type 2 diabetes risk in a Chinese Han population. Am J Clin Nutr [Internet]. 2012 Mar;95(3):626–32. Available from: http://www.ncbi.nlm.nih.gov/pubmed/22301935

47. Batar B, Bavunoglu I, Hacioglu Y, Cengiz M, Mutlu T, Yavuzer S, et al. The role of TMPRSS6 gene variants in iron-related hematological parameters in Turkish patients with iron deficiency anemia. Gene [Internet]. 2018;673(January):201–5. Available from: https://doi.org/10.1016/j.gene.2018.06.055

48. Nai A, Pagani A, Silvestri L, Campostrini N, Corbella M, Girelli D, et al. TMPRSS6 rs855791 modulates hepcidin transcription in vitro and serum hepcidin levels in normal individuals. Blood [Internet]. 2011 Oct 20;118(16):4459–62. Available from: http://www.bloodjournal.org/cgi/doi/10.1182/blood-2011-06-364034

49. Danquah I, Gahutu J-B, Zeile I, Musemakweri A, Mockenhaupt FP. Anaemia, iron deficiency and a common polymorphism of iron-regulation, TMPRSS6 rs855791, in Rwandan children. Trop Med Int Health [Internet]. 2014;19(1):117–22. Available from: http://www.ncbi.nlm.nih.gov/pubmed/24175968

50. Pei SN, Ma MC, You HL, Fu HC, Kuo CY, Rau KM, et al. TMPRSS6 rs855791 polymorphism influences the susceptibility to iron deficiency anemia in women at reproductive age. Int J Med Sci. 2014;11(6):614–9.

51. Beutler E, Van Geet C, te Loo DMWM, Gelbart T, Crain K, Truksa J, et al. Polymorphisms and mutations of human TMPRSS6 in iron deficiency anemia. Blood Cells, Mol Dis [Internet]. 2010 Jan 15;44(1):16–21. Available from: http://www.ncbi.nlm.nih.gov/pubmed/19818657

52. Pelusi S, Girelli D, Rametta R, Campostrini N, Alfieri C, Traglia M, et al. The A736V TMPRSS6 polymorphism influences hepcidin and iron metabolism in chronic hemodialysis patients: TMPRSS6 and hepcidin in hemodialysis. BMC Nephrol [Internet]. 2013;14:48. Available from: http://www.pubmedcentral.nih.gov/articlerender.fcgi?artid=3585892&tool=pmcentrez&rendertype=abstract

53. Ganesh SK, Zakai NA, van Rooij FJA, Soranzo N, Smith A V, Nalls MA, et al. Multiple loci influence erythrocyte phenotypes in the CHARGE Consortium. Nat Genet [Internet]. 2009 Nov 11;41(11):1191–8. Available from: http://dx.doi.org/10.1038/ng.466

54. van der Harst P, Zhang W, Mateo Leach I, Rendon A, Verweij N, Sehmi J, et al. Seventy-five genetic loci influencing the human red blood cell. Nature. 2013;492(7429):369–75.

55. Bédard A, Lewis SJ, Burgess S, John Henderson A, Shaheen SO. Maternal iron status during pregnancy and respiratory and atopic outcomes in the offspring: A Mendelian randomisation study. BMJ Open Respir Res. 2018;5(1):1–10.

56. Kamatani Y, Matsuda K, Okada Y, Kubo M, Hosono N, Daigo Y, et al. Genome-wide association study of hematological and biochemical traits in a Japanese population. Nat Genet [Internet]. 2010;42(3):210–5. Available from: http://dx.doi.org/10.1038/ng.531

57. Tanaka T, Roy CN, Yao W, Matteini A, Semba RD, Arking D, et al. A genome-wide association analysis of serum iron concentrations. Blood [Internet]. 2010 Jan 7;115(1):94–6. Available from: http://www.ncbi.nlm.nih.gov/pubmed/19880490

58. Valenti L, Rametta R, Dongiovanni P, Motta BM, Canavesi E, Pelusi S, et al. The A736V TMPRSS6 Polymorphism Influences Hepatic Iron Overload in Nonalcoholic Fatty Liver Disease. PLoS One. 2012;7(11).

59. Cheng HL, Hancock DP, Rooney KB, Steinbeck KS, Grif HJ, Connor HTO. SHORT COMMUNICATION A candidate gene approach for identifying differential iron responses in young overweight women to an energy-restricted haem iron-rich diet. 2014;(February):1250–2.

60. Seiki T, Naito M, Hishida A, Takagi S, Matsunaga T, Sasakabe T, et al. Association of genetic polymorphisms with erythrocyte traits: Verification of SNPs reported in a previous GWAS in a Japanese population. Gene [Internet]. 2018;642(October 2017):172–7. Available from: http://dx.doi.org/10.1016/j.gene.2017.11.031

61. Alfred T, Ben-Shlomo Y, Cooper R, Hardy R, Deary IJ, Elliott J, et al. Genetic variants influencing biomarkers of nutrition are not associated with cognitive capability in middle-aged and older adults. J Nutr [Internet]. 2013 May;143(5):606–12. Available from: http://jn.nutrition.org/cgi/doi/10.3945/jn.112.171520

62. Ji Y, Flower R, Hyland C, Saiepour N, Faddy H. Genetic factors associated with iron storage in Australian blood donors. Blood Transfus. 2018;16(2):123–9.

63. Guo MH, Nandakumar SK, Ulirsch JC, Zekavat SM, Buenrostro JD, Natarajan P, et al. Comprehensive population-based genome sequencing provides insight into hematopoietic regulatory mechanisms. Proc Natl Acad Sci [Internet]. 2017;114(3):E327–36. Available from: http://www.pnas.org/lookup/doi/10.1073/pnas.1619052114

64. Jackson HA, Carter K, Darke C, Guttridge MG, Ravine D, Hutton RD, et al. HFE mutations, iron deficiency and overload in 10 500 blood donors. Br J Haematol. 2001;114(2):474–84.
