# Supplementary material for: Differences in the frequency of genetic variants associated with iron imbalance among global populations
Source: PLoS One. 2020 Jul 1;15(7):e0235141. doi: 10.1371/journal.pone.0235141 (PMC7329092; doi:10.1371/journal.pone.0235141)
Supplement: S3 Table — (DOCX) [file pone.0235141.s004.docx]

**S4 Table: Population Branch Statistic (PBS) values involving the comparison of three populations**.

|  |  |  |  |  |  |
| --- | --- | --- | --- | --- | --- |
|  | **SNP** | **PBS-AFR** | **PBS-EUR** | **PBS-SAS** |  |
|  | **rs10421768** | 0.001 | 0.003 | 0.000 |  |
|  | **rs744653** | 0.001 | 0.003 | 0.000 |  |
|  | **rs11568350** | 0.021 | 0.000 | 0.000 |  |
|  | **rs1439816** | 0.266 | 0.051 | 0.000 |  |
|  | **rs2235321** | 0.000 | 0.003 | 0.027 |  |
|  | **rs855791** | 0.163 | 0.000 | 0.071 |  |
|  | **rs78174698** | 0.000 | 0.011 | 0.051 |  |
|  | **rs5756504** | 0.109 | 0.000 | 0.060 |  |
|  | **rs5756506** | 0.249 | 0.000 | 0.078 |  |
|  | **rs4820268** | 0.045 | 0.000 | 0.043 |  |
|  | **rs2413450** | 0.116 | 0.000 | 0.057 |  |
|  | **rs2072860** | 0.098 | 0.000 | 0.035 |  |
|  | **rs9610643** | 0.098 | 0.000 | 0.035 |  |
|  | **rs855788** | 0.286 | 0.000 | 0.042 |  |
|  | **rs2543519** | 0.031 | 0.009 | 0.000 |  |
|  | **rs2111833** | 0.000 | 0.002 | 0.025 |  |
|  | **rs2235324** | 0.000 | 0.001 | 0.002 |  |
|  | **rs1421312** | 0.017 | 0.013 | 0.000 |  |
|  | **rs5756512** | 0.011 | 0.010 | 0.000 |  |
|  | **rs2160906** | 0.026 | 0.013 | 0.000 |  |
|  | **rs732756** | 0.017 | 0.012 | 0.000 |  |
|  | **rs228904** | 0.016 | 0.012 | 0.000 |  |
|  | **rs11704654** | 0.001 | 0.002 | 0.000 |  |
|  | **rs5756516** | 0.000 | 0.030 | 0.027 |  |
|  | **rs228916** | 0.027 | 0.006 | 0.000 |  |
|  | **rs228918** | 0.001 | 0.000 | 0.006 |  |
|  | **rs228921** | 0.001 | 0.000 | 0.006 |  |
|  | **rs1867504** | 0.089 | 0.000 | 0.009 |  |
|  | **rs9872999** | 0.000 | 0.029 | 0.026 |  |
|  | **rs8177179** | 0.000 | 0.024 | 0.022 |  |
|  | **rs12493168** | 0.047 | 0.019 | 0.000 |  |
|  | **rs1799852** | 0.034 | 0.000 | 0.017 |  |
|  | **rs1799899** | 0.033 | 0.005 | 0.000 |  |
|  | **rs3811658** | 0.093 | 0.000 | 0.021 |  |
|  | **rs8177248** | 0.130 | 0.000 | 0.024 |  |
|  | **rs8177253** | 0.032 | 0.000 | 0.014 |  |
|  | **rs1405023** | 0.040 | 0.000 | 0.013 |  |
|  | **rs1880669** | 0.054 | 0.013 | 0.000 |  |
|  | **rs3811647** | 0.042 | 0.000 | 0.014 |  |
|  | **rs1358024** | 0.125 | 0.000 | 0.033 |  |
|  | **rs1525892** | 0.014 | 0.000 | 0.010 |  |
|  | **rs1049296** | 0.032 | 0.000 | 0.028 |  |
|  | **rs7638018** | 0.071 | 0.000 | 0.019 |  |
|  | **rs1830084** | 0.093 | 0.000 | 0.019 |  |
|  | **rs2280673** | 0.000 | 0.010 | 0.027 |  |
|  | **rs1799945** | 0.043 | 0.043 | 0.000 |  |
|  | **rs1800562** | 0.001 | 0.020 | 0.000 |  |
|  | **rs198846** | 0.000 | 0.013 | 0.012 |  |
|  | **rs129128** | 0.052 | 0.026 | 0.000 |  |
|  | **rs7385804** | 0.000 | 0.003 | 0.000 |  |
|  | **Average** | 0.053 | 0.008 | 0.017 |  |
|  |  |  |  |  |  |

(AFR=Africa, EUR- Europe, SAS=South Asia)

|  |  |  |  |
| --- | --- | --- | --- |
